# Targeting Transporters for Drug Delivery to the Brain: Can We Do Better?

**DOI:** 10.1007/s11095-022-03241-x

**Published:** 2022-03-31

**Authors:** Elena Puris, Gert Fricker, Mikko Gynther

**Affiliations:** Institute of Pharmacy and Molecular Biotechnology, Ruprecht-Karls-University, Im Neuenheimer Feld 329, 69120 Heidelberg, Germany

**Keywords:** CNS, drug delivery, nanocarriers, prodrugs, transporter

## Abstract

Limited drug delivery to the brain is one of the major reasons for high failure rates of central nervous system (CNS) drug candidates. The blood–brain barrier (BBB) with its tight junctions, membrane transporters, receptors and metabolizing enzymes is a main player in drug delivery to the brain, restricting the entrance of the drugs and other xenobiotics. Current knowledge about the uptake transporters expressed at the BBB and brain parenchymal cells has been used for delivery of CNS drugs to the brain via targeting transporters. Although many transporter-utilizing (pro)drugs and nanocarriers have been developed to improve the uptake of drugs to the brain, their success rate of translation from preclinical development to humans is negligible. In the present review, we provide a systematic summary of the current progress in development of transporter-utilizing (pro)drugs and nanocarriers for delivery of drugs to the brain. In addition, we applied CNS pharmacokinetic concepts for evaluation of the limitations and gaps in investigation of the developed transporter-utilizing (pro)drugs and nanocarriers. Finally, we give recommendations for a rational development of transporter-utilizing drug delivery systems targeting the brain based on CNS pharmacokinetic principles.

## Introduction

Central nervous system (CNS) diseases including neurological disorders and brain cancers, remain to be one of the major causes of disability and death worldwide (1). Despite an alarming increase in incidence over the last few years, CNS diseases lack treatments. One of the reasons for high failure rates in CNS drug development is limited access of drugs to the target site in the brain. In order to produce a pharmacological effect, a drug needs to cross the blood–brain barrier (BBB) in sufficient concentration and interact with a target within the brain. The BBB, composed of brain capillary endothelial cells with their tight junctions, multiple transporters, receptors and metabolizing enzymes, strictly regulates the passage of the molecules in order to avoid the entrance of harmful xenobiotics to the brain. The high defensive properties and complex physiology of the BBB make the brain the most inaccessible organ in terms of drug delivery. On the other hand, to maintain CNS function, the BBB allows passage of nutrients to the brain and elimination of metabolites and/or other potentially harmful molecules to the blood or cerebrospinal fluid (CSF). Many essential compounds such as glucose and amino acids can enter the brain via transporters expressed at the membrane of the brain endothelial cells (2).

Knowledge about transporters expressed at the BBB and structural requirements for substrate binding and translocation via the transporters have been used for development of transporter-mediated drug delivery systems targeting the brain via specific transporter proteins. The strategy includes 2 processes: (i) transporter-mediated permeation of a drug itself, or in form of a prodrug or nanocarrier through the BBB and in some cases the cellular barrier of brain parenchyma; and (ii) release of the active (parent) drug from a prodrug or nanocarrier at the site of action inside the brain.

More than a hundred attempts to develop transporter-utilizing (pro)drugs and nanoparticles/liposomes (hereinafter referred as nanocarriers) have been made to improve the uptake of CNS drugs into the brain. However, the majority of the (pro)drugs and nanocarriers has not reached clinical trials. This fact raises the question about the effectiveness of the approach and its further potential to be used in humans. In the present review, we provide a systematic summary of the current progress in development of transporter-utilizing (pro)drugs and nanocarriers for delivery of drugs into the brain. We critically evaluate the effectiveness of the approach and give recommendations for evaluation of brain delivery efficacy by transporter-utilizing delivery systems based on CNS pharmacokinetic principles. In addition, the advantages and limitations of the approach as well as future perspectives and gaps in our knowledge about the approach are discussed.

## Structure of the Blood–Brain Barrier and Neurovascular Unit

The main role of the CNS consisting of the brain and spinal cord is to regulate normal functioning of the body through integrating and coordinating input signals and providing a proper response. In this respect, the maintenance of the homeostasis of the CNS microenvironment is vital (3) and achieved by the presence of 3 main cellular barriers at the blood-CNS interfaces such as the BBB, the blood-cerebrospinal fluid barrier (BCSFB) and the arachnoid barrier (4) regulating nutrient flux, clearance of waste products, immune cell distribution and limiting the access of toxicants and pathogens.

**Fig. 1:**
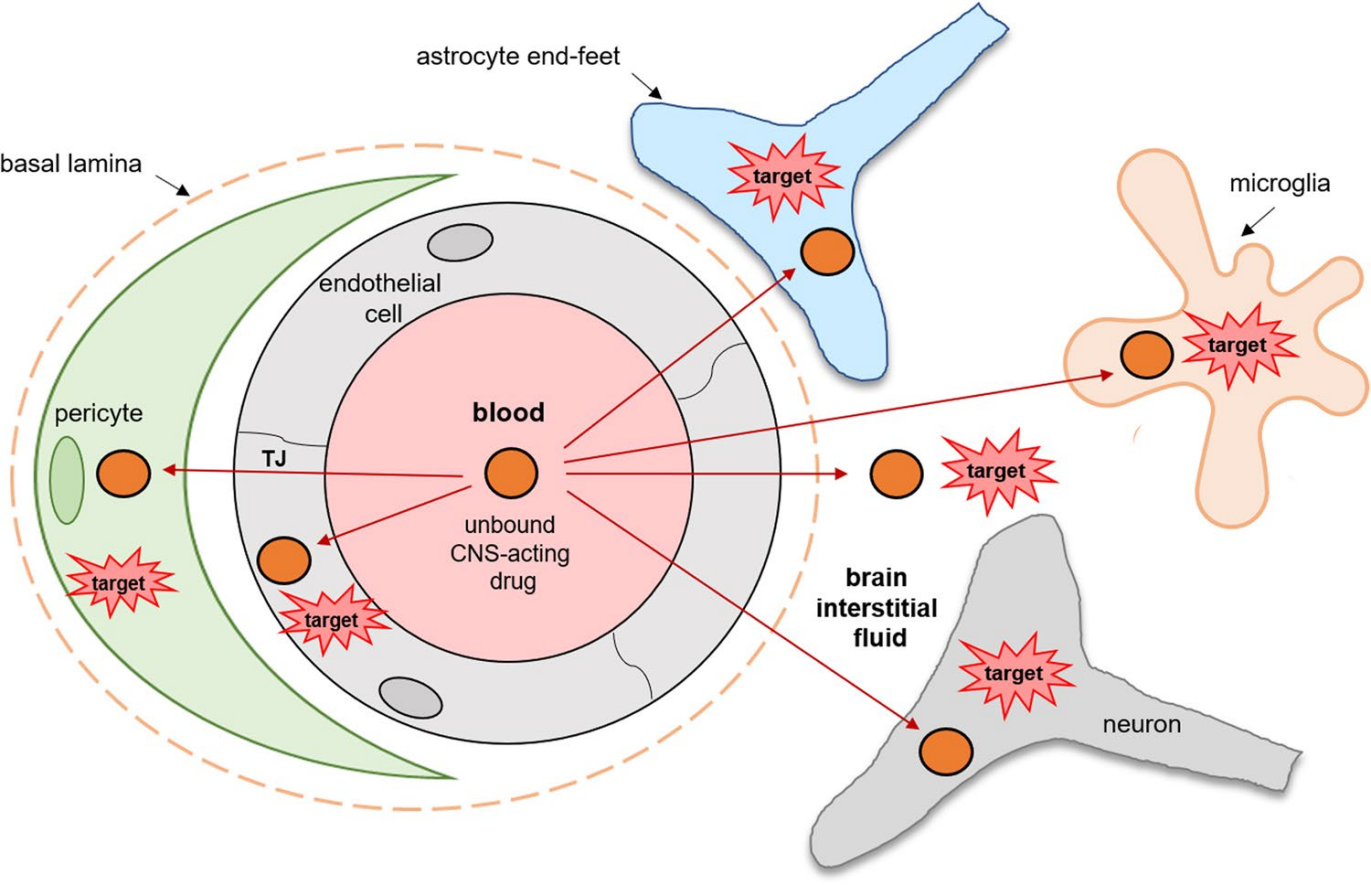
The schematic representation of the blood–brain barrier with neurovascular unit cells and delivery of unbound CNS drug from blood to the target site in the brain. TJ – tight junctions.

The BBB separates the blood and the brain interstitial fluid (ISF, also referred to extracellular fluid, ECF), and it is a key player in drug delivery to the brain (Fig. 1). The reason lies in an extensive blood capillary network, with an average surface area of the cerebral microvessels of 100 – 200 cm^2^ per gram of adult human brain (5, 6) available for the passage of compounds between the blood and brain parenchyma. Importantly, the parenchymal cells surrounded by ECF are easily accessible due to their close location. Thus, the distance from neurons to the nearest cerebral microvessel is about 10–20 μm (7). The restrictive properties of the BBB as a “physical” barrier are characterized by the lack of fenestrations and the presence of tight junctions (TJ) between the brain capillary endothelial cells (8, 9). TJs are formed by transmembrane proteins such as occludin, claudins, and junctional adhesion molecules (10). These proteins interact with their counterparts on adjacent cell plasma membranes and integral proteins, forming a fence restricting paracellular diffusion of polar substances, lipids (11, 12). TJs are involved in close contact with the actin skeleton, adherens junctions and perivascular cells such as astrocytes and pericytes (13). Moreover, TJs create and control the polarization of the brain capillary endothelial cells (14).

The BBB is a dynamic barrier, and its protective function is achieved by the presence of metabolizing enzymes, multiple membrane transporters and receptors, as well as interaction with the brain parenchymal cells accounting for 80% of the brain tissue volume (15). The brain cells, i.e. pericytes, perivascular astrocytic end-feet, mast cells, microglia and immune cells together with the BBB form the so-called neurovascular unit (NVU) (16). The close communication between the NVU cells is controlled by multiple signalling systems and provides a strong mechanism of brain defence (17). Additionally, the brain parenchymal cells express efflux transporters and metabolizing enzymes protecting them from toxic and harmful substances (18). Furthermore, the ISF bulk flow facilitating the elimination of substances to the CSF allows the maintenance of the microenvironment (19). The alteration of the brain microenvironment can result in impaired protective properties of the BBB and can lead to CNS diseases (16, 20).

## Transport Mechanisms Across the Blood–Brain Barrier

**Fig. 2:**
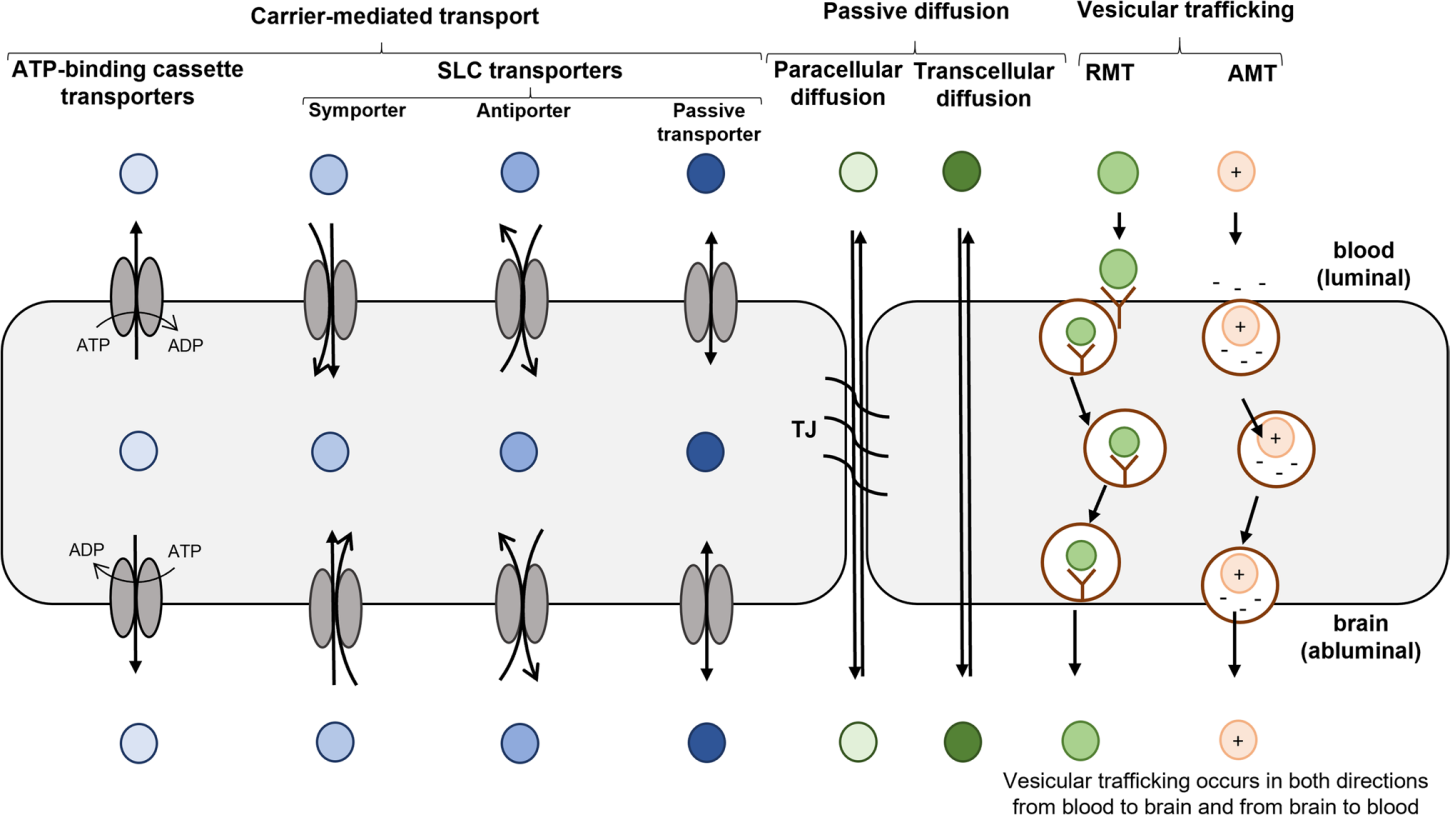
The transport routes across the BBB. Passive diffusion, passive solute carrier (SLC) mediated delivery, receptor-mediated transcytosis (RMT) and adsorptive-mediated transcytosis (AMT) occur in both directions from blood to brain and from brain to blood.

Despite the restricted entry of compounds to the brain, there are several routes by which essential nutrients and other molecules, including drugs, can permeate the BBB (Fig. 2). They include passive diffusion, vesicular trafficking, and carrier-mediated transport (CMT).

### Passive Diffusion

Passive diffusion (Fig. 2) is an energy-independent process driven by the unbound compound concentration gradient on both sides of the endothelial cell membrane, which is proportional to the diffusion rate (21). Transport can occur between the endothelial cells (paracellular) or through the cells (transcellular). Due to the presence of TJs, the paracellular passive diffusion at the BBB is negligible. In contrast, highly lipophilic compounds with small molecular sizes, such as opioids and diazepam, can permeate the BBB through transcellular passive diffusion, which has been considered for a long time as the main route by which compounds can permeate the BBB. As some physicochemical properties of the compounds showed correlation between the BBB permeability via passive diffusion (22–24), their improvement has been used during CNS drug development. For instance, as an increase in drug lipophilicity resulted in a proportionally higher BBB permeation rate, many drug delivery strategies were focused on an increase in lipophilicity of the CNS drugs (25). The effect of the increased drug lipophilicity on BBB permeation can be attributed to greater nonspecific brain tissue binding in the brain parenchyma, leading to concentration gradients, which drive the transcellular passive diffusion (26). Importantly, as only unbound drugs can interact with the target in the brain, increased nonspecific brain tissue binding can affect unbound drug concentrations in the brain. In addition, more lipophilic drugs will tend to pass not only through the BBB, but also to other organs, which consequently results in a reduced availability of drugs to enter the brain (27). Increased lipophilicity of the drug may lead to enhanced plasma protein binding resulting in a diminished unbound plasma concentration of the drug (28). Therefore, improved lipophilicity as a strategy for drug delivery to the brain should be considered with caution.

### Vesicular Trafficking

Large molecular weight compounds, such as peptides and proteins, required for normal brain functioning can pass through the BBB via transcytosis (Fig. 2), which can be specific and mediated by binding to a particular receptor or non-specific, such as cationization and adsorptive-mediated transcytosis (Fig. 2).

*Receptor-mediated transcytosis (RMT)* occurs in 3 phases: endocytosis, intracellular vesicular trafficking, and finally exocytosis (29). Endocytosis is a major component of cells adapting their protein composition on the plasma membrane. Molecules capable of binding to membrane proteins can trigger the internalization of the protein and simultaneously protein-bound molecules (30, 31). Receptor-mediated endocytosis is also known as clathrin-dependent endocytosis as the membrane-associated protein clathrin takes part in forming membrane vesicles that become internalized into the cell (32). After the formation of a ligand-receptor complex, it diffuses laterally in the plasma membrane until encountering a coated pit, where the receptor-ligand complex accumulates with clathrin, adaptor protein, and dynamin. This is followed by curving of the membrane and formation of an internalized clathrin-coated vesicle. After the protein–ligand complex is invaginated and intracellular transport vesicles are formed, clathrin and dynamin recycle back to the plasma membrane and the vesicles are converged in the early endosome network (33). The early endosome network is responsible for intracellular sorting from which the protein–ligand complex can be transported via sorting tubules for exocytosis, thus completing the transcytosis. However, the vesicles containing the protein–ligand complex can also be fused with lysosomes leading to the intracellular release of the ligand. The fate of the protein–ligand complex during sorting is independent on the protein, the type of ligand binding to the receptors, and the type of cell in which the protein–ligand complex is internalized (34). In addition to the physiological role of receptor-mediated transcytosis, such as the brain delivery of leptin via leptin receptors (35), the phenomenon has been harnessed for the delivery of drugs across the BBB and is considered as one of the most promising brain drug delivery strategies; the advances in this field have been recently reviewed elsewhere (36, 37).

*Adsorptive-mediated transcytosis (AMT)* is a non-selective process with a lack of targeting specificity. It is driven by nonspecific electrostatic interactions between cationic proteins, for instance histone and avidin, and anionic microdomains on endothelial cells (38–40). The carboxyl groups of glycoproteins forming the glycocalyx on the luminal endothelial cell membrane provide anionic sites for the binding of cationic molecules allowing cellular internalization (39). The process is initiated by binding of positively charged moieties of macromolecules to the negatively charged plasma membrane of the brain capillary endothelial cells (41). The current knowledge about the AMT mechanisms at BBB as well as utilization of AMT for CNS drug delivery are described in a review by Hervé *et al.* (2008) (39).

In addition to RMT and AMT, *transcytosis of transporter-binding nanocarriers* has been investigated and utilized in order to deliver drugs into the brain. The transporter-mediated nanocarrier delivery of drugs into the brain is discussed in the present review.

### Carrier-Mediated Transport (CMT)

CMT of small molecules across the BBB occurs after the interaction between a substrate compound and the specific transport protein, which can be expressed in either the luminal or abluminal membrane or both sides of the brain capillary endothelial cells (Table I). The expression and function of the transporters at the brain capillary endothelial cells determine whether the passage of molecules is unidirectional referring to either influx or efflux across the BBB (Fig. 2) or bidirectional denoting to the passage of solutes in both directions across the BBB according to the concentration gradient (42).

**Table I:** Characteristics of Transporters which Absolute Protein Expression was Quantified at Human Brain Capillaries and Examples of their Substrates

Gene name (Protein name)	Transport type	Localization in plasma membrane	Substrates	Protein expression fmol/µg total protein (mean ± SD)
ABC transporters				
ABCA2 (ABC2)	ATP-binding cassette	Not known	Cholesterol, estrodiol, estramustine	2.86 ± 0.58 (50) 2.11 ± 0.78 (51) 0.08 ± 0.03 (52)
ABCA8 (ABCA8)	ATP-binding cassette	Not known	Organic anions	1.21 ± 0.24 (50) 0.67 ± 0.23 (51)
ABCB1 (P-gp, MDR1)	ATP-binding cassette	Luminal	Vincristine, quinidine, verapamil	6.06 ± 1.69 (50) 3.98 ± 0.88 (51) 2.58 ± 0.93 (52)
ABCC4 (MRP4)	ATP-binding cassette	Luminal and abluminal	E_2_17βG, methotrexate, topotecan	0.195 ± 0.069 (50) 0.31 ± 0.11 (51)
ABCC8 (SUR1)	ATP-binding cassette	Luminal	Tolbutamide, glibenclamide, diazoxide, pinacidil	0.277 ± 0.047 (50)
ABCG2 (BCRP)	ATP-binding cassette	Luminal	Glutathione, folic acid, mitoxantrone, topotecan, dantrolene	8.14 ± 2.26 (50) 6.15 ± 1.41 (51) 2.22 ± 0.61 (52)
SLC transporters				
SLC1A2 (EAAT2)	C/Na^+^, H^+^, K^+^	Abluminal	L-glutamine, D/L- aspartic acid	5.7 ± 1.9 (51)
SLC1A3 (EAAT1)	C/Na^+^, H^+^, K^+^	Abluminal	L-glutamine, D/L- aspartic acid	24.5 ± 12.5 (50) 30.72 ± 13.07 (51)
SLC1A4 (ASCT1)	C/Na^+^, E/amino acids	Abluminal	L-alanine, L-serine, L-cysteine, L-threonine	1.81 ± 0.53 (51)
SLC2A1 (GLUT1)	F	Luminal and abluminal	Glucose, galactose, mannose, glucosamine	139 ± 46 (50) 78.5 ± 23 (51) 21.9 ± 9.80 (52)
SLC2A3 (GLUT3)	F	Luminal and abluminal	Glucose, galactose, mannose, xylose	4.40 ± 1.00* (50) 2.53 ± 0.73* (51)
SLC6A12 (BGT1)	C/Na^+^, Cl^−^	Not known	Betaine, GABA	3.16 ± 0.94 (50) 2.89 ± 0.81 (51)
SLC7A1 (CAT1)	F (non-obligatory E)	Luminal	Cationic L-amino acids	1.13 ± 0.18 (50) 0.99 ± 0.34 (51)
SLC7A5 (LAT1)	E	Luminal and abluminal	Large neutral amino acids, L-dopa, gabapentin T3, T4	0.431 ± 0.091 (50) 0.8 ± 0.25 (51) 0.59 ± 0.15 (52)
SLC16A1 (MCT1)	C/H^+^ or E/monocarboxylate	Luminal and abluminal	Lactate, pyruvate, ketone bodies	2.27 ± 0.85 (50) 1.46 ± 0.39 (51) 5.37 ± 3.73 (52)
SLC16A2 (MCT8)	F	Luminal and abluminal	T2, rT3, T3, T4	1.31 ± 0.37 (51)
SLC19A1 (RFC)	E/organic phosphates	Not known	Reduced folates, antifolates	0.763 ± 0.041 (50) 0.55 ± 0.18 (51)
SLCO1A2 (OATP1A2)	Not known	Luminal	Bile salts, organic anions and cations	0.54 ± 0.10 (52)
SLCO1B3 (OATP8)	Not known	Not known	Bile salts, organic anions	0.46 ± 0.15 (52)
SLCO2B1 (OATP2B1)	Not known	Luminal and abluminal	Estrone–3-sulfate, dehydroepiandrosterone	0.40 ± 0.04 (52)
SLCO1C1 (OATP1C1)	Not known	Luminal and abluminal	T4, T3, rT3	0.27 ± 0.03 (52)
SLC22A1 (OCT1)	F	Luminal	Organic cations	0.58 ± 0.11 (52)
SLC22A3 (OCT3)	F	Luminal	Organic cations	0.62 ± 0.08 (52)
SLC22A6 (OAT1)	E/organic anions	Not known	Organic anions	0.48 ± 0.11 (52)
SLC22A7 (OAT2)	F or E	Luminal	Organic anions	7.90 ± 3.80 (52)
SLC22A8 (OAT3)	E/dicarboxylate	Abluminal	Organic anions	0.27 ± 0.03 (52)
SLC22A9 (OAT7)	E/short chain fatty acids	Not known	Organic anions	0.51 ± 0.10 (52)
SLC27A1 (FATP1)	LCFA transport, VLCFA activation	Abluminal	LCFA, VLCFA	2.08 ± 0.38 (51)
SLC29A1 (ENT1)	F	Luminal	Nucleosides, nucleotides, nucleobases	0.568 ± 0.134 (50) 0.86 ± 0.13 (51) 0.27 ± 0.10 (52)

^*^ The protein expression refers to the total amount of SLC2A3 and SLC2A14

ABC transporters—ATP binding cassette transporters; ASCT—Alanine/Serine/Cysteine-preferring transporter; BCRP—Breast cancer resistance protein; BGT—Sodium- and chloride-dependent betaine transporter; C – Co-transporter; EAAT—Excitatory amino acid transporter; ENT—Equilibrative nucleoside transporter; E – Exchanger; F – Facilitated transporter; FATP—Fatty acid transporter protein; GLUT—Glucose transporter; LAT1—Large neutral amino acid transporter; LCFA – Long-chain fatty acids; MCT—Monocarboxylic acid transporter; MDR1—Multidrug resistance protein 1; MRP—Multidrug resistance-associated protein; OAT: Organic anion transporter; OATP—Organic-anion-transporting polypeptide; OCT—Organic cation transporter; P-gp—P-glycoprotein; RFC—Reduced folate carrier; rT3 – Reverse triiodothyronine; SUR1—Sulfonylurea receptor; T3 – Triiodothyronine, T4 – Thyroxine, VLCFA – Very long-chain fatty acids

The information about transporter type and substrates is based on previous publications (53–70)

Depending on the mechanism and use of the energy source, the process of transporter-mediated uptake is classified as passive (also known as facilitated or equilibrative transport) or active transport. While passive transporters, such as glucose transporter, enable delivery of solutes across the cellular membrane along their electrochemical gradient without energy consumption, active transporters generate solute gradients across the membranes and exploit various energy-coupling mechanisms. Active transporters are divided into primary or secondary carriers based on the source of cellular energy used to drive the process. Primary active transporters such as the adenosine triphosphate (ATP)-binding cassette (ABC) transporters and ion pumps (ATPases) efflux solutes from the cell or into the organelles against their concentration gradient utilizing the energy of the hydrolytic reaction of ATP (43, 44). Ion pumps actively transporting ions such as H^+^, Na^+^, K^+^, Ca^2+^ and Cu^2+^ produce and maintain electrochemical ion gradients across the cellular membranes which are subsequently used by secondary active ion-coupled transporters to deliver molecules in antiport (also known as exchange) or symport (also known as co-transport) of these ions (45, 46). In addition to ions, other solutes can also be co-transported. The stoichiometry of solute(s) or ion(s) passage via transporters is fixed per translocation cycle. CMT is a temperature-dependent and saturated process following Michaelis–Menten kinetics, which can be affected by various types of inhibitors, which include competitive (competing for transport-active binding site), non-competitive (modulating substrate binding allosterically), uncompetitive (hampering the modification of the substrate-transporter complex conformation) or mixed-type inhibitors (47).

Most transporters expressed at the BBB belong to 2 major superfamilies such as ABC transporters or solute carriers (SLCs) which possess differences in substrate translocation. Protein expression of several SLC and ABC transporters has been quantified at isolated human brain capillary endothelial cells by quantitative absolute proteomics. Information about these transporters and their substrates is given in Table I. However, one should remember that transporter expression does not guarantee its functional activity.

The transporters mediate not only the BBB passage of essential nutrients such as glucose, amino acids, and vitamins, but also the influx and efflux of the CNS drugs to and out of the brain (Table I), thereby influencing the concentration of drugs at the target site within the brain. Importantly, the transporters, which are expressed at the membrane of brain parenchymal cells, mediate the intracellular distribution of drugs within the brain, while transporters, expressed at the BBB, and BCSFB, influence elimination of the therapeutics (48, 49).

Efflux transporters expressed at the BBB limit the brain access of such drugs, which are substrates of these transporters. For instance, efflux transporters such as ABCB1, ABCG2, and ABCC4 are considered to be a major factor limiting drug delivery to the brain (50). In contrast, SLC transporters mediate the brain influx of drugs across the BBB (48). Several SLC transporters expressed at the BBB and brain parenchymal cells have been used for development of transporter-utilizing (pro)drugs or nanocarriers for delivery to the brain.

## Transporter-mediated approach for brain delivery of drugs

**Fig. 3:**
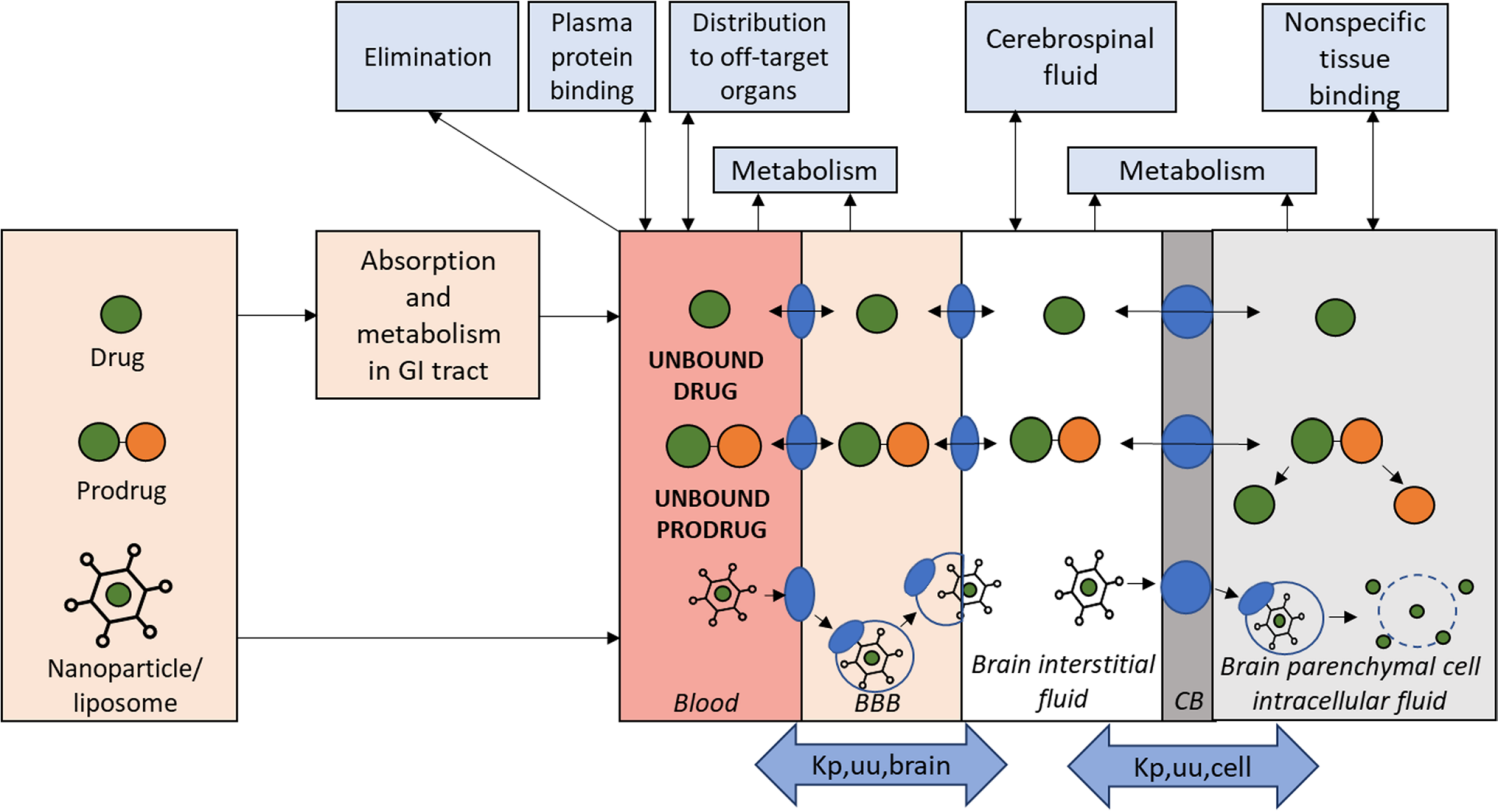
A schematic illustration of the main pharmacokinetic principles of transporter-mediated drug delivery to the brain after the administration of either drug, prodrug or nanocarrier including transport across the blood–brain barrier (BBB) and brain parenchymal cellular barrier (CB).

Great efforts have been taken to deliver drugs into the brain, which resulted in the development of several drug delivery strategies (67–70). One of the strategies, which has drawn increasing attention over the recent years, is a transporter-mediated drug delivery approach. This strategy is based on the delivery of the drug from systemic circulation across the BBB via specific transporters expressed at the luminal and abluminal sites of the brain capillary endothelial cells and, in case of the drugs with intracellular targets, at the brain parenchymal cells (Fig. 1, 3). Thus, first, a drug should be influxed from the blood to the brain capillary endothelial cells via a luminal uptake transporter and subsequently effluxed to the brain ECF via an abluminal transporter. For drugs with intracellular targets, it is important that after reaching the brain ECF, the drug crosses the cellular membrane of the brain parenchymal cells via a transporter expressed in the target cells.

The transporter-mediated drug delivery approach includes 3 main strategies: (i) modification of the drug structure in a way that a specific BBB transporter will recognize it as a substrate, e.g. “pseudo nutrient”, and deliver the drug across the cellular membrane; (ii) conjugation of the parent drug with a promoiety representing nutrient-substrate of the particular BBB transporter known as a prodrug strategy; (iii) decoration of the drug-loaded nanocarriers with a moiety representing substrate of the particular BBB transporter. The knowledge about functional expression of transporters at the BBB and structural requirements for substrate binding and translocation via the transporters gives tempting opportunity for rational design and development of drugs, prodrugs and nanocarriers, which can utilize the specific BBB transporter for brain delivery of CNS drugs (71, 72).

### Transporter-Mediated Prodrug Approach

The transporter-mediated prodrug approach is based on delivery of the drug in the form of a prodrug, representing a substrate of the transporter, across the luminal and abluminal membranes of the brain capillary endothelial cells as well as the brain parenchymal cellular barrier followed by the release of the parent drug at the target site within the brain (Fig. 3). Prodrugs per se are therapeutically inactive (73), undergo enzymatic and/or chemical bioconversion, and release active parent drug at the target site within the brain. Importantly, the release of the parent drug *in vivo* should occur in a predictable manner. In case of the transporter-mediated prodrug approach, the rational design of prodrugs should take into account the functional expression and structural requirements for substrate binding and translocation via the transporter, as well as capability to be bioconverted by an enzyme at the target site (74). This requires extensive knowledge about the processes driven by a particular transporter and metabolizing enzyme. Thus, in terms of development of transporter-utilizing prodrugs for delivery to the brain, it is essential to understand the mechanism of BBB permeation, intra-brain distribution and release of parent drug from prodrug at the target site within the brain (Fig. 3).

In order to cross the cellular membrane, the transporter utilizing prodrugs must have molecular features for efficient transporter binding. The structural requirements for efficient binding to the targeted transporter are crucial, particularly, when the prodrug has to compete against endogenous substrates, such as amino acids or glucose, having high concentrations in the body (75, 76). In addition, the prodrugs must have structural features for effective translocation across the cellular membrane via the targeted transporter, as, otherwise, they would act more as inhibitors than substrates (77). Furthermore, for the efficient transporter-mediated permeation across the BBB, the prodrugs must be able to bind also to the intracellular substrate binding site of the transporter at the abluminal membrane of the brain capillary endothelial cells and to be translocated out of the endothelial cells into the ECF and, subsequently, to the brain parenchyma through the targeted transporter (Figs. 2, 3). However, it has been proven challenging to design transporter-utilizing prodrugs which fulfill all the forementioned properties. For example, large neutral amino acid transporter 1 (LAT1)-utilizing amino acid prodrugs have been shown to bind to LAT1 in high affinity but the transport rates into the cells were dramatically lower compared to endogenous substrates, L-amino acids (78, 79). Importantly, there is limited information available about the LAT1-targeted prodrug binding to the intracellular binding site, which is a prerequisite for the efflux of the produgs from endothelial cells into the brain parenchyma. For prodrugs targeting other SLC transporters, such as Glucose transporter 1 (GLUT1), sodium-dependent vitamin C transporter 2 (SVCT2) and Organic cation/carnitine transporter 2 (OCTN2), there is even less information available as the transport kinetics have not been investigated for prodrugs targeted to these transporters.

### Transporter-Mediated Nanocarrier Delivery Approach

The transporter-mediated nanocarrier delivery strategy is based on the incorporation of transporter substrates on the nanocarrier surface, which bind to the transporter protein on the cellular membrane and trigger the internalization process (80). For transporter-mediated drug delivery into the intracellular compartment of the brain parenchyma, nanocarriers must first be transported through the BBB by transcytosis followed by endocytosis into the target cells where the nanocarrier releases its cargo (Fig. 3).

The specific mechanism leading to transporter protein-nanocarrier complex internalization is dependent on the targeted membrane protein. For example, LAT1-targeted nanocarriers bind to the LAT1 and lock the transporter in an occluded conformation, which results in the inhibition of cellular LAT1-mediated amino acid uptake. The mechanism of LAT1-nanocarrier complex internalization is likely induced by the inhibition of amino acid influx into the cell. The resulting low amino acid concentration in the cell leads to reduced mTOR signaling and activates a ubiquitination-dependent mechanism promoting the endocytosis and recycling of LAT1 (81). In a study by Li *et al.* (2016), LAT1-targeted nanoparticles reduced the plasma membrane expression of the transporter in HeLa cells when measured 1 h after exposure (82). The colocalization of LAT1 and Rab11 after 1 h incubation showed that after the nanoparticle binding, LAT1 entered into Rab11-positive recycling endosomes (82). The plasma membrane expression of LAT1 was regained after 3 h of nanoparticle exposure, indicating that the transporter had dissociated from the nanoparticle and relocated on the cell membrane, leaving the nanoparticle to be degraded in lysosomes. Unfortunately, there are currently no studies showing the transcytosis mechanism of LAT1-targeted nanocarriers across the BBB. GLUT1 undergoes continuous recycling and internalization proceeds through both clathrin-dependent and -independent endocytosis pathways (83). After the fusing of GLUT1-containing vesicles with Early Endosome Antigen 1-positive vesicles, they are either delivered to the lysosome for degradation or recycled back to the membrane surface (83). Recently, Wang *et al.* (2021) reported that a GLUT1-targeted nanodisk underwent endocytosis by clathrin-mediated endocytosis and energy-dependent macro-pinocytosis pathway (84). However, the mechanisms which trigger GLUT1 internalization after nanocarrier binding remain elusive. OCTN2 is another transporter which has been investigated for the delivery of drugs via nanocarriers. He *et al.* (2020) studied the ability of carnitine-coated micelles to utilize OCTN2 for transcytosis across Caco-2 cell monolayers (85). The micelles underwent clathrin-mediated endocytosis, delivery to lysosomes followed by the escaped micelle transport to the endoplasmic reticulum and Golgi apparatus. In addition, the authors reported that the micelles were transported through Caco-2 cell monolayers via caveolae/lipid rafts-mediated mechanism and were transported directly to the endoplasmic reticulum and Golgi apparatus after being endocytosed by the cells, followed by exocytosis. In another study by Kou *et al.* (2017), carnitine conjugated nanoparticles were reported to undergo caveolin-mediated, clathrin-mediated endocytosis and micropinocytosis in Caco2 cells (86). However, there are no studies available to determine whether the transport of OCTN2 utilizing nanocarriers across the BBB follows similar processes as across the Caco-2 monolayers.

## Pharmacokinetic Concepts for Evaluation of Drug Delivery Into the Brain

The development of transporter-mediated brain delivery systems is complex, and evaluation of the efficacy of the delivery to the brain is complicated due to the unique physiology and anatomy of the brain and its barriers. In general, the assessment of the brain delivery efficacy of the developed transporter-utilizing (pro)drugs and nanocarriers has to be done based on the CNS pharmacokinetic principles in order to provide accurate information about the effectiveness of the approach.

The estimation of target‐site concentrations of the drug in the brain is vital for understanding of the pharmacodynamic response. Importantly, only unbound drugs can pass the cellular membrane, undergo biotransformation (in the case of prodrugs), and interact with a target. Therefore, the evaluation of the efficacy of the delivery via transporter-mediated approach should be performed with the main focus on unbound concentration of the active (parent) drug at the specific site in the brain where the target is located.

In general, a (pro)drug or nanocarrier administered by a specific route follows numerous pharmacokinetic processes such as absorption (with exception to intraarterial and intravenous administration), distribution, metabolism, and excretion (Fig. 3). These processes can affect the bioavailability of the administered (pro)drug or nanocarrier and consequently its delivery to the target site in the brain. Along with the delivery to the brain, the drug can also be distributed to other organs where it may cause side effects. Similarly, prodrugs or nanocarriers can release parent drug after distribution to off-target organs leading to toxic effects. Therefore, targeting of the (pro)drugs and nanocarriers via transporters to the brain and release of the parent drug from the prodrug and nanocarriers at their site of action is desirable, although difficult to achieve in practice.

The drug delivery to the brain is characterized by three main neuropharmacokinetic determinants, such as rate of BBB permeation, extent of brain delivery and intra-brain distribution of drug, allowing to assess the brain delivery efficacy of developed transporter-utilizing (pro)drugs or nanocarriers. The detailed explanation of the CNS pharmacokinetic concepts and evaluation of these parameters can be found in previously published review (87).

### BBB Permeation Rate

The BBB permeation rate provides an estimate of the unidirectional transport of a drug across the BBB, which is different from the extent of equilibrated drug transport across the BBB at steady state. The rate of brain permeation indicates how fast is the process of brain drug delivery across the BBB. Several techniques have been developed for the evaluation of the BBB such as the *in vivo* brain uptake index (BUI) method (88), *in situ* brain perfusion (89), cerebral microdialysis technique (90, 91), and cell uptake studies in the *in vitro* models of the BBB (92). The rate of permeation determined by efflux index method is usually described by efflux clearance (*Cl*_out_), which represents the net passage out of the brain and includes passive and active BBB transport as well as metabolism in the brain and elimination to the CSF by bulk flow (91). Other methods, for example *in situ* brain perfusion technique, allow determination of the *Cl*_in_, which is based on evaluation of the unidirectional transfer constant (*K*_in_) and permeability surface area product (PS product). Both parameters describe measurements of clearances, rather than rates per se (87)*.* In chronic treatment, the rate of BBB permeation is not as important as the extent of drug delivery.

### Extent of the BBB Transport

The extent of the BBB transport, described by the drug amount or concentration in the brain at steady state in relation to blood levels of the drug, is an important characteristic of drug delivery to the brain. In general, the brain delivery extent (Fig. 3) is estimated by the ratio of total concentrations in the brain and plasma (K_p,brain_) or the ratio of unbound concentrations in the brain ISF and plasma (K_p,uu,brain_) (87). The K_p,brain_ values are influenced by both the BBB transport and plasma/brain tissue binding. Due to the fact that only unbound drugs can cross the BBB, K_p,uu,brain_ provides more relevant information about the net flux across the BBB, as a combination of the influx and efflux transport across the BBB. Thus, when K_p,uu,brain_ is close to unity, it indicates that drug BBB delivery is dominated by passive processes (91). If to assume the low influence of metabolism in the brain and low ISF bulk flow, K_p,uu,brain_ larger than unity will display a dominating active influx of the drug to the brain ISF, while K_p,uu,brain_ lower than unity will indicate a dominating active efflux from the brain (93–96). The standard method for direct estimation of the brain delivery extent (K_p,uu,brain_) is a cerebral microdialysis technique, which is used for continuous monitoring of unbound concentrations of the drug in the brain ISF and blood over the time via inserted probes into the brain parenchyma (90). In addition, the extent of brain delivery can be measured by monitoring drug concentrations in the blood and brain after an intravenous (i.v.) or intraperitoneal (i.p.) injection. However, the method is restricted due to the use of one individual brain tissue and blood concentration measurements at terminal sampling. The obtained areas under the total concentration–time curve for drug in the brain and plasma (AUC_total_) are used for calculation of K_p,brain_ or can be combined with unbound fraction determined by equilibrium dialysis to calculate K_p,uu,brain_ using a combinatory mapping approach (87, 97). In addition, imaging methods including positron emission tomography (PET) and single-photon emission computed tomography (SPECT) have been developed and applied for estimation of drug delivery to the brain over the time *in vivo* (98). However, due to the limited availability and high costs of the radiotracers, the application of imaging techniques for investigation of the brain delivery extent has been restricted.

### Intra-Brain Distribution

After crossing the BBB via specific transporter, a drug, prodrug or nanocarrier can distribute from the brain ISF into the brain parenchymal cells via the same transporter expressed at the cellular membrane followed by the release of parent drug (in case of prodrugs and nanocarriers) or binding to the cell constituents, e.g. phospholipids or cellular proteins (Fig. 3). The intra-brain distribution of compounds is generally described by the unbound volume of distribution in the brain (V_u,brain_, mL/g brain), which can be estimated using an in *in vitro* brain slice method (87, 99–101). The V_u,brain_ describes the relationship between the total concentration of the drug in the brain and its unbound concentration in the brain ISF (87). In respect of physiological volumes of the brain fluids, V_u,brain_ of 0.2 mL/g brain is the lowest possible value, which is equal to ISF volume, V_brain,ISF_ (102), while V_u,brain_ close to the 0.8 mL/g brain displays the water volume in the brain indicating an even distribution within the brain (103). In contrast, V_u,brain_ greater than 0.8 mL/g brain demonstrates the predominant distribution to the brain parenchymal cells. As the transporter-utilizing (pro)drugs and nanocarriers are distributed in and out of the brain parenchymal cells via transporters expressed at cellular membranes, the ISF concentration of the unbound (pro)drug or nanocarriers is not necessarily equal to that in the brain parenchymal cells (49). Moreover, the distribution to the brain parenchymal cells can be cell-specific depending on the expression of the particular transporter at the membrane of the individual cell. As a direct measurement of unbound concentrations of drugs (C_u,cell_) inside the brain parenchymal cells cannot be achieved in practice, indirect methods combining the brain slice assay and brain homogenate method have been established to estimate C_u,cell_ (97, 104). The method is based on the assumption, that the binding of the (pro)drug occurs mainly inside the cells and that estimation of C_u,cell_ indicates the overall unbound drug concentration in the brain intracellular compartment without specifying the distribution to the specific parenchymal cells or subcellular structures. Moreover, unbound volume of distribution in the brain parenchymal cells (V_u,cell_) estimated using the brain homogenate method and cerebral microdialysis can be combined with V_u,brain_ to estimate unbound (pro)drug concentration ratio in intra- and extracellular compartments of the brain (K_p,uu,cell_) (87). The parameter describes the unbound (pro)drug distribution between the brain ISF and brain parenchymal cellular compartment representing an average ratio for all parenchymal cells at steady-state. K_p,uu,cell_ higher than unity characterises the intracellular distribution of the (pro)drugs, while K_p,uu,cell_ lower than unity describes preferable extracellular distribution within the brain.

## Current status of transporter-mediated approach for drug delivery to the brain

One of the factors limiting development of drug delivery to the brain via a transporter-mediated approach is the complexity of evaluation of brain delivery efficacy of drugs. Here, we critically evaluate and discuss advances in development of currently reported transporter-utilizing (pro)drugs and nanocarriers in light of the main CNS pharmacokinetic principles.

### Selection of Drug and Transporter

The need for the improvement of drug delivery to the brain arises from an insufficient efficacy of drug treatments. The development of transporter-utilizing (pro)drugs and nanocarriers for improved brain delivery begins from the understanding whether the poor efficacy of the drug is caused by low extent of the brain permeation and inadequate concentrations of the drug at target site or due to other reasons. Second, information about the localization of the drug target in the brain, which can be extra- or intracellular, as well as functional expression of the selected transporter in the brain is essential for the selection of relevant transporter to be targeted at the BBB and brain parenchymal cells. Thus, targeting of the transporters expressed only at the BBB would be useful in terms of delivery of drugs with extracellular targets in the brain, while utilization of the transporters expressed at the BBB and cellular membrane of the brain parenchymal cells is essential for delivery of the drugs with intracellular targets. Among reviewed studies, the following SLC transporters have been targeted for delivery of various drugs to the brain: organic cation/carnitine transporter 2 (OCTN2), glucose transporters (GLUTs), LAT1, sodium-dependent vitamin C transporter 2 (SVCT2) and glutathione transporters (Tables II and III).

**Table II:** (Pro)drugs Developed to Utilize SLC Transporters for Improvement of Brain Delivery of Drugs

(Pro)drug	Investigation of prodrug activity	Evidence of utilization of specific transporter	Information about the BBB permeation and extent of brain delivery	Information about intra-brain distribution	In vivo pharmacodynamic evidence	Clinical evidence
LAT1						
Isoleucinyl ester of the 5’-hydroxyl group of zidovudine (105, 106)	Similar or more potent in inhibiting the viral replication in vitro compared to parent drug	NR	In vivo PK study in rabbits: after i.v. injection of 18 mg/kg prodrug C_30min,total,brain_/C_30min,total,plasma_ of released parent drug at was same compared to parent drug dosing (10 mg/kg), C_75min,total,brain_/C_75min,total,plasma_ of released parent drug was twice higher compared to parent drug dosing	NR	NR	NR
L-4-chlorokynurenine (4-Cl-KYN), a prodrug of 7-chlorokynurenic acid (107, 108)	NR	In situ brain perfusion in rats: saturated BBB uptake V_max_ 16.9 ± 2.3 nmol/min/g and K_m_ of 105 ± 14 µM 4-Cl-KYN reduced L-[^14^C]leucine uptake to the brain in a concentration-dependent manner (Ki 116 ± 10 µM)	In situ brain perfusion in rats: 4-Cl-KYN crossed the BBB and released parent drug 20 s after perfusion with 100 µM or 500 µM prodrug Cerebral microdialysis study in rats: release of parent drug in hippocampus within 1 h after prodrug administration with increased release until a steady state was reached at about 3 h	NR	NR	NR
Tyrosine prodrug of nipecotic acid (109)	NR	NR	NR	NR	In vivo study in genetically seizure-prone strain (DBA/2) of mice: prodrug showed significant dose-dependent anticonvulsant activity after i.p. injection, while parent drug had no anticonvulsant effect	NR
L-cysteine conjugate of 2-methyl-1-propanethiol (110)	NR	In situ brain perfusion in rats: prodrug inhibited [^14^C]L-leucine brain uptake by 92% Prodrug K_i_ of [^14^C]L-leucine uptake in different brain regions ranged 6.9–12.8 µM	NR	NR	NR	NR
L-cysteine conjugate of 6-mercaptopurine (110)	NR	In situ brain perfusion in rats: prodrug inhibited [^14^C]L-leucine uptake by 63%	NR	NR	NR	NR
L-tyrosine prodrug of ketoprofen (111)	NR	In situ brain perfusion in rats: prodrug inhibited [^14^C]L-leucine brain uptake by 98% Prodrug brain uptake K_m_ was 22.5 ± 9.18 μM and V_max_ = 1.4 ± 0.15 pmol/mg/min Prodrug brain uptake was significantly inhibited by BCH	In situ brain perfusion in rats: prodrug crossed BBB, the concentration of prodrug in the endothelial cell-enriched pellet fraction was below the lower limit of detection	NR	NR	NR
Lysine derivative of ketoprofen (79, 112–114)	Active prodrugs, as demonstrated by inhibition of COX peroxidase activity in vitro (IC50 1.05 µM)	In situ rat brain perfusion: prodrug inhibited [^14^C]L-leucine brain uptake by 79%, prodrug brain uptake K_m_ 231.6 ± 60.4 μM and V_max_ 1.50 ± 0.20 pmol/mg/min, brain uptake of prodrug was significantly inhibited by L-phenylalanine In vitro uptake study: In ARPE19 cells K_m_ 6.9 μM and V_max_ 11.1 pmol/min/mg protein Prodrug inhibited [^14^C]L-leucine uptake by 17.6% Prodrug uptake was inhibited by LAT1 inhibitor by 61.5%	In situ brain perfusion in mice: BBB rate of permeation K_in_ 0.06 Cerebra microdialysis study in rats: K_p,uu,brain_ prodrug 0.09 K_p,uu,brain_ ketoprofen 0.12 K_p,uu,brain_ released ketoprofen 0.33 In vivo PK study in mice: AUC_u,brain_/AUC_u,plasma_ 0.016 after a single dose of 25 μmol/kg i.p, no detected parent drug in the brain	Cerebral microdialysis study in rats: K_p,uu,cell_ of prodrug 1.2, while for parent drug—0.47 K_p,uu,cell_ for released parent drug—63.6 Cerebral microdialysis study in mice: K_p,uu,cell_ of prodrug 0.03, while for parent drug – not determined (lower detection limit) K_p,uu,cell_ for released parent drug—63.6	NR	NR
Ester-based meta- and para-substituted phenylalanine prodrugs of valproic acid (115, 116)	NR	In situ brain perfusion in rats: prodrugs inhibited [^14^C]L-leucine brain uptake (K_i_ of meta- and para-substituted prodrugs 2.7 μM, and 32.4 μM, respectively), In vitro uptake in MCF7 cells: no detectable uptake of prodrugs, prodrugs inhibited 78% and 46% of [^14^C]L-leucine, respectively	In situ brain perfusion in rats: the BBB permeation rate of prodrugs (600 μM) was 4.7 and 3.1 pmol/mg/min, respectively	NR	NR	NR
Amide-based meta- and para-substituted phenylalanine prodrugs of valproic acid (115, 116)	NR	In situ brain perfusion in rats: prodrugs inhibited [^14^C]L-leucine brain uptake (K_i_ of meta- and para-substituted prodrugs 3.2 μM, and 34.1 μM, respectively), In vitro uptake in MCF7 cells: prodrugs inhibited 81% and 48% of [^14^C]L-leucine, respectively Cellular uptake for meta- and para-substituted prodrugs were ca 28 pmol/min/mg protein	In vivo PK study in rats: after i.v. bolus injection of 40 μmol/kg of meta-substituted prodrug K_p,brain_ prodrug 0.069 K_p,brain_ of released parent drug from prodrug 0.056, after i.v. bolus injection of 40 μmol/kg of para-substituted prodrug K_p,brain_ prodrug 0.039, no detected parent drug in the brain K_p,brain_ parent drug dosing 0.048 In situ brain perfusion in rats: the BBB permeation rate of prodrugs (600 μM) was 28.9 and 12.2 pmol/mg/min, respectively	NR	NR	NR
Amide-based para-substituted phenylalanine prodrug of valproic acid with additional methylene linker (115)	NR	In vitro uptake in MCF7 cells: prodrug inhibited 89% of [^14^C]L-leucine Cellular uptake of prodrug was 48 pmol/min/mg of protein	In vivo PK study in rats: after i.v. bolus injection of 40 μmol/kg prodrug K_p,brain_ prodrug 0.24 and K_p,brain_ released parent drug 0.3 while for parent drug K_p,brain_ 0.048	NR	NR	NR
L-lysine-methotrexate conjugate (117)	NR	NR	In vivo PK study in mice: after i.v. injection 7.15 mM/kg of prodrug or parent drug K_p,brain_ released parent drug 0.34 K_p,brain_ parent drug 0.08 AUC_total,brain_ of released parent drug was 7.74 times higher after prodrug dosing compared to parent drug itself	NR	NR	NR
Aspartic acid prodrug of dopamine and 2-amino-apidic acid prodrug of dopamine (118)	NR	In situ brain perfusion in rats: 35–38% inhibition of [^14^C]L-leucine brain uptake by prodrugs	In situ brain perfusion in rats (only 2-amino-apidic acid prodrug of dopamine): Prodrug was not detected in the brain after perfusion	NR	NR	NR
Meta-conjugated phenylalanine prodrug of dopamine (118, 119)	NR	In situ brain perfusion in rats: 85% inhibition of [^14^C]L-leucine brain uptake by prodrugs, the BBB uptake K_m_ 227 μM and V_max_ 0.99 pmol/mg/min Prodrug brain uptake was inhibited by 61% by L-phenylalanine In vitro study in MCF7 cells: concentration-dependent inhibition of [^14^C]L-leucine cellular uptake by prodrug (12.5–100 μM)	In situ brain perfusion in rats: prodrug crossed BBB after perfusion In vivo PK study in rats: after i.p. injection of prodrug K_p,brain_ prodrug 0.009, no improvement of dopamine levels in brain after prodrug administration compared to L-dopa or control animals	NR	NR	NR
L-tyrosine carbamate prodrug of dopamine (119)	NR	In vitro study in MCF7 cells: prodrug inhibited [^14^C]L-leucine cellular uptake. IC50 was calculated with four concentrations of prodrug	NR	NR	NR	NR
Ester-based and amide-based prodrugs of perforin inhibitors (120–123)	NR	In situ brain perfusion in mice: 97% and 59% inhibition of [^14^C]L-leucine brain uptake by ester-and amine-based prodrugs, respectively Brain uptake of ester-based prodrug was inhibited by co-treatment of probenecid and L-tryptophan, brain uptake amide-based prodrug was inhibited by probenecid alone and probenecid plus L-tryptophan In vitro study in MCF7 cells: ester-based prodrug K_m_ 37 µM and V_max_ 10 nmol/mg/min Amide-based prodrug K_m_ 13 µM and V_max_ 0.60 nmol/mg/min In astrocytes: [^14^C]L-leucine brain uptake inhibition by ester-based prodrug (IC50 3.5 μM), not saturable uptake at 1–200 μM prodrug	In vivo PK study in mice: after i.p. bolus injection of 23 μmol/kg ester- and amide-based prodrugs i.p. in mice K_p,brain_ ester-based prodrug was 0.043, K_p,brain_ amide-based prodrug was 0.035 Released parent drug was detected only after ester-based prodrug dosing Parent drugs itself were not detected in the brain after the dosing	In vitro study in mouse cortical astrocytes and cortical neurons: improved uptake of prodrugs in mouse astrocytes and neurons compared to parent drugs In vitro study in mouse primary neurons and astrocytes, as well as immortalized microglia: Concentration-dependent uptake in mouse primary neurons, astrocytes, and microglia at concentration 25, 50, 100 μM	In vivo study in LPS-induced inflammation mouse model: no improvement of reduction of prostaglandin E2 levels in the brain after prodrug dosing compared to parent drug dosing	NR
Meta-conjugated phenylalanine derivative of ketoprofen (79, 113, 122, 124)	Activity of prodrug to inhibit COX peroxidase activity was not sufficiently studied, as only limited concentrations of prodrug were investigated compared to ketoprofen and other prodrugs	In vitro study in ARPE19 cells: prodrug uptake K_m_ 8.9 μM, V_max_ 62.4 pmol/min/mg protein, Prodrug inhibited 91% of [^14^C]L-leucine cellular uptake Prodrug uptake was inhibited by LAT1 inhibitor by 88% In vitro study in mouse primary neurons and astrocytes, as well as immortalized microglia: Prodrug cellular uptake was significantly inhibited by LAT1 inhibitor in neurons, astrocytes, and microglia	In situ brain perfusion in mice: rate of BBB permeation of prodrug K_in_ 0.36 µL/s/g In vivo PK study in mice: after a single dose of 25 μmol/kg prodrug i.p.: AUC_u,brain_/AUC_u,plasma_ 0.008 for prodrug, AUC_u,brain_/AUC_u,plasma_ 0.13 for released parent drug, while AUC_u,brain_/AUC_u,plasma_ of ketoprofen 0.01 after parent drug dosing	In vitro brain slice method in mice: K_p,uu,brain_ 0.006 for prodrug compared to K_p,uu,brain_ 0.042 for parent drug, V_u,brain_ prodrug 24 mL/g brain and K_p,uu,cell_ of prodrug 1.4 V_u,brain_ parent drug 1.5 mL/g brain, K_p,uu,cell_ 0.19 for parent drug In vitro brain slice method in rats: V_u,brain_ prodrug 31 mL/g brain, K_p,uu,cell_ 1.9 for prodrug V_u, brain_ parent drug 1.8 mL/g brain, K_p,uu,cell_ 0.24	In vivo study in LPS-induced inflammation mouse model: no improvement of reduction of prostaglandin E2 levels in the brain after prodrug dosing compared to parent drug dosing	NR
Para-conjugated phenylalanine prodrug of ketoprofen (79)	NR	In vitro study in ARPE19 cells: prodrug uptake K_m_ 7.6 μM and V_max_ 68.4 pmol/min/mg protein, Prodrug inhibited 76% of [^14^C]L-leucine cellular uptake Prodrug uptake inhibited by LAT1 inhibitor by 65.9%	In situ brain perfusion in mice: rate of BBB permeation of prodrug K_in_ 0.31 µL/s/g In vivo PK study in mice: after a single dose of 25 μmol/kg prodrug i.p.: AUC_u,brain_/AUC_u,plasma_ 0.009 for prodrug, AUC_u,brain_/AUC_u,plasma_ 0.35 of released parent drug, while AUC_u,brain_/AUC_u,plasma_ 0.01 of ketoprofen after parent drug dosing	NR	NR	NR
Meta-conjugated phenylalanine prodrug of ketoprofen with additional methylene linker (79)	NR	In vitro study in ARPE19 cells: prodrug uptake K_m_ 3.8 μM and V_max_ 16.7 pmol/min/mg protein Prodrug inhibited 88% of [^14^C]L-leucine cellular uptake Prodrug uptake inhibited by LAT1 inhibitor by 72%	In situ brain perfusion in mice: rate of BBB permeation of prodrug K_in_ 0.29 µL/s/g In vivo PK study in mice: after a single dose of 25 μmol/kg prodrug i.p.: AUC_u,brain_/AUC_u,plasma_ 0.001 for prodrug, no detected parent drug in the brain	NR	NR	NR
Ketoprofen derivative with aliphatic amino acid promoiety (79, 113)	Active prodrug, as demonstrated by inhibition of COX peroxidase activity in vitro (IC50 2.26 µM)	In vitro study in ARPE19 cells: prodrug uptake K_m_ 19.8 μM and V_max_ 110 pmol/min/mg protein Prodrug inhibited 6.2% of [^14^C]L-leucine cellular uptake Prodrug uptake inhibited by LAT1 inhibitor by 63%	In situ brain perfusion in mice: rate of BBB permeation of prodrug K_in_ 0.45 µL/s/g In vivo PK study in mice: after a single dose of 25 μmol/kg prodrug i.p.: AUC_u,brain_/AUC_u,plasma_ 0.35 for prodrug, no detected parent drug in the brain after prodrugs’ dosing	NR	NR	NR
Ester-based meta-conjugated phenylalanine derivative of ketoprofen (113, 122, 125)	Active prodrugs, as demonstrated by inhibition of COX peroxidase activity in vitro (IC50 0.58 µM)	In vitro study in MCF7 cells: prodrug uptake V_max_ 0.24 nmol/min/mg protein and K_m_ 49.2 µM Uptake of prodrug was not inhibited by LAT1 inhibitor, but by probenecid sensitive inhibitor In vitro study in mouse primary neurons and astrocytes, as well as immortalized microglia: prodrug uptake was significantly inhibited by LAT1 inhibitor in neurons, astrocytes, and microglia	NR	In vitro study in mouse primary neurons and astrocytes, as well as immortalized microglia: Concentration-dependent uptake in mouse primary neurons, astrocytes, and microglia at concentration 25, 50, 100 μM	NR	NR
L-lysine apigenin carbamate (126)	NR	NR	In vivo PK study in mice: after i.p. administration of 0.4 mg/g prodrug, parent drug was detected in the brain, while after parent drug dosing 0.23 mg/g no parent drug were not detected	NR	NR	NR
Amide-based meta-conjugated phenylalanine prodrug of ferulic acid with an additional methylene linker (122, 127)	NR	In vitro study in ARPE19 cells: prodrug uptake K_m_ 24 μM, V_max_ 4.5 pmol/min/mg protein Prodrug inhibited 86% of [^14^C]L-leucine cellular uptake Prodrug uptake inhibited by LAT1 inhibitor by 65% In vitro study in mouse primary neurons and astrocytes, as well as immortalized microglia: prodrug uptake was significantly inhibited by LAT1 inhibitor in neurons, astrocytes, and microglia	In situ brain perfusion in mice: rate of BBB permeation of prodrugs K_in_ 2.8 µL/s/g In vivo PK study in mice: after a single dose of 25 μmol/kg prodrug i.p.: K_p,brain_ prodrug 0.32, no released parent drug in the brain	In vitro study in mouse primary neurons and astrocytes, as well as immortalized microglia: Concentration-dependent uptake in mouse primary neurons, astrocytes, and microglia at concentration 25, 50, 100 μM	NR	NR
Amide based meta-conjugated phenylalanine prodrug of ferulic acid (122, 127)	NR	In vitro study in ARPE19 cells: prodrug uptake K_m_ 16 μM, V_max_ 1.8 pmol/min/mg protein Prodrug inhibited 62% of [^14^C]L-leucine cellular uptake Prodrug uptake inhibited by LAT1 inhibitor by 72% In vitro study in mouse primary neurons and astrocytes, as well as immortalized microglia: prodrug uptake was significantly inhibited by LAT1 inhibitor only in astrocytes	In situ brain perfusion in mice: rate of BBB permeation of prodrug K_in_ 1.91 µL/s/g In vivo PK study in mice: K_p,brain_ prodrug 1.24 K_p,brain_ released parent drug 0.02, while K_p,brain_ parent drug 0.01 after parent drug dosing	In vitro study in mouse primary neurons and astrocytes, as well as immortalized microglia: Concentration-dependent uptake in mouse primary neurons, astrocytes, and microglia at concentration 25, 50, 100 μM	NR	NR
Ester-based phenylalanine prodrug of ferulic acid (122, 127)	NR	In vitro study in ARPE19 cells: Prodrug inhibited 55% of [^14^C]L-leucine cellular uptake, prodrug uptake was not inhibited by LAT1 inhibitor In vitro study in mouse primary neurons and astrocytes, as well as immortalized microglia: prodrug was not detected in neurons and microglia at concentration < 10 µM, prodrug uptake was not inhibited by LAT1 inhibitor in astrocytes	NR	In vitro study in mouse primary neurons and astrocytes, as well as immortalized microglia: Concentration-dependent uptake in mouse primary neurons, astrocytes, and microglia at concentration 25, 50, 100 μM	NR	NR
Prodrug of dopamine conjugated via a secondary carbamate linker to L-tyrosine (119)	NR	In vitro study in MCF7 cells: concentration-dependent inhibition of [^14^C]L-leucine cellular uptake by prodrug 12.5–100 µM No saturation of the uptake at concentrations 3.1–100 µM	NR	NR	NR	NR
Prodrugs of efflux inhibitor probenecid (128, 129)	NR	In vitro study in MCF7 cells: prodrugs with aromatic promoieties inhibited [^14^C]L-leucine cellular uptake, while the aliphatic amino acid conjugate did not do The uptake of amide-based meta-conjugated phenylalanine prodrug was inhibited by L-tryptophan	NR	NR	In vivo PK study in mice: after co-administration of prodrug and vinblastine i.p. 25 µmol/kg, no improvement of brain delivery extent of efflux transporter substrate vinblastine, as K_p,brain_ of vinblastine was 0.05, while after co-administration with prodrug K_p,brain_ of vinblastine was 0.048	NR
Ester-based prodrugs of flurbiprofen, ibuprofen, naproxen and ketoprofen (130)	NR	In vitro study in mouse immortalized microglia and primary cortical astrocytes: Prodrugs inhibited [^14^C]L-leucine cellular uptake in microglia and astrocytes, respectively: IC50 of flurbiprofen prodrug 4.2 and 7.3 µM IC50 of ibuprofen prodrug 5.3 and 23 µM IC50 of naproxen prodrug 10 and 15 µM IC50 of ketoprofen prodrug 15 and 112 µM Reported K_m_ values of prodrugs’ uptake are higher than the measured concentrations	NR	NR	NR	NR
Glucose transporters						
L-serinyl β-D-glucoside analogues of [Met^5^]enkephalin (131)	NA	NR	NR	NR	Analgesia effects in mice after i.p. administration of the compound	NR
Glucose-chlorambucil derivative (132)	NR	Inhibition of the uptake of [^14^C]D-glucose into human erythrocytes by derivative (IC50 0.065 ± 0.015 mM) Concentration-dependent inhibition of [^3^H]cytochalasin B binding to erythrocytes	NR	NR	NR	NR
D-glucose ester prodrug of 7-chlorokynurenic acid (133, 134)	Prodrug was not active in vitro, as demonstrated by absence of inhibition of [^3^H]glycine binding	NR	Cerebral microdialysis study in rats: 1000-fold higher levels of prodrug and parent drug in cortical perfusate 2 h after i.p. injections of equal dose of prodrug and parent drug 200 mg/kg No plasma concentration reported	In vitro study in mouse cortical cultures containing both neurons and astrocytes: uptake and bioconversion of prodrug to parent drug	In vivo study in mice: Protective effect against seizures induced NMDA in mice after i.p. injection of prodrug 200 mg/kg with anticonvulsive activity, while parent drug did not show efficacy	NR
Glycosyl prodrugs of dopamine (135)	Prodrugs were not active as they did not inhibit the binding of [^3^H]spiperone to D_2_ receptor	Inhibition of the uptake of [^14^C]D-glucose into human erythrocytes by prodrugs (IC50s 12.1—100 mM)	NR	NR	In vivo study in reserpinized mice: no antiparkinsonian properties were revealed for the prodrugs	NR
Glycosyl prodrugs of dopamine (136)	NR	Inhibition of the uptake of [^14^C]D-glucose into human erythrocytes by prodrugs (IC50s 1.5—100 mM)	NR	NR	NR	NR
Glycose and galactose derivative of dopamine and L-dopa (137, 138)	NR	Inhibition of the uptake of [^3^H]3-*O-*methylglucose in HRPE by glycosyl dopamine prodrug coupled via a succinic space (IC50 2.6 ± 0.6 mM) The uptake of the prodrug was inhibited by 10 mM D-glucose	NR	NR	In vivo study of effect on morphine induced locomotion in mice: glycosyl prodrug of L-dopa was more potent in reducing morphine-induced locomotion than L-dopa or galactose derivative of L-dopa In vivo study in reserpinized rats: glycosyl and galactose derivative of dopamine derivatives showed similar efficacy in reversing hypolocomotion, and were more active than L-dopa or ester glycosyl and galactose prodrugs of L-dopa	NR
Glycosyl prodrug of GW196771 (139)	NR	NR	In vivo PK study in rat: improvement of brain penetration after i.v. injection of 1 mg/kg dose of prodrug compared to parent compound	NR	NR	NR
Dopamine-gluconamine (IPX-750) and dopamine-gluconamide (IPX-760) (140)	IPX-750 and IPX-760 bind and activates D1/D5 receptors in vitro	NR	NR	NR	In vivo study in Parkinson’s disease models, MPTP-lesioned mice and *Nurr1(*+ */ −)* knockout mouse and 6-OHDA lesioned rats: Improved locomotor performance after IPX-750 treatment	NR
Ketoprofen-glucose prodrug, indomethacin-glucose prodrug (141)	NR	In situ brain perfusion in rats: inhibition of BBB uptake of 0.2 μCi/mL [^14^C]D-glucose by ketoprofen and indomethacin prodrugs (IC50s 33.0 ± 8.2 μM 0.71 ± 0.04 µM, respectively) Reduction of the brain uptake of ketoprofen prodrug by 61.4% and no effect on indomethacin prodrug uptake after co-perfusion with 50 mM with D-glucose	In situ brain perfusion in rats: rate of BBB permeation of 150 μM ketoprofen prodrug 1.3 ± 0.18 pmol/mg/min, rate of BBB permeation of 150 μM indomethacin prodrug 1.9 ± 0.43 pmol/mg/min	NR	NR	NR
Glycosyl thiamine disulfide prodrugs of naproxen with lock-in function (142)	NR	NR	In vivo PK study in mice: more than 1.7-fold higher AUC_0-240 min,brain_ after i.v. injection of 10 mg/kg of prodrugs, K_p,brain_ of released parent drug ranged 0.36–0.42, while for parent drug itself 0.49	NR	NR	NR
Naproxen prodrug conjugated to glucose (143)	NR	NR	In vivo PK study in mice: 2.0 times higher AUC_0-480 min,brain_ and 1.9 times higher C_max_ of total released parent drug after i.v. administration of prodrug compared to parent drug itself	NR	In vivo study in cerebral ischemia rat model: Increased neuroprotective effect after prodrug administration compared to parent drug itself	NR
Venlafaxine-thiamine disulfide-glucose prodrug (V-TDS-G) and venlafaxine-glucose prodrug (V-G) (144)	NR	NR	In vivo PK study in mice: after i.v. injection of 10 mg/kg K_p,brain_ of released parent drug from V-TDS-G was 1.19, K_p,brain_ of released parent drug from V-G was 0.44, K_p,brain_ of parent drug after venlafaxine dosing was 0.24	NR	NR	NR
Ibuprofen prodrug conjugated to glucose (145)	In vitro study: not significant neuroprotective effect in H_2_O_2_-induced oxidative stress and injury PC12 cells treated with prodrug	NR	In vivo PK study in mice: 2.1 times higher AUC_0-480 min,brain_ and 4.1 times higher C_max_ of total released parent drug after i.v. administration of 10 mg/kg prodrug compared to parent drug itself K_p,brain_ of released parent drug 0.51, while for parent drug itself 0.34	NR	In vivo cerebral ischemia rat model: greater neuroprotective properties after i.v. administration of prodrug compared to parent drug	NR
Ester-based prodrugs of ibuprofen conjugated to D-glucose (146)	NR	NR	In vivo PK study in rats: After i.v. injection of 18 mg/kg of prodrugs or 10 mg/kg of parent drug, K_p,brain_ of released parent drug was 0.19—0.57 compared to K_p,brain_ 0.58 after parent drug dosing	NR	NR	NR
SVCT2						
Naproxen prodrug conjugated to ascorbic acid (143)	NR	NR	In vivo PK study in mice: 2.1 times higher AUC_0-480 min,brain_ and 2.2 times higher C_max_ of total released parent drug after i.v. administration of prodrug compared to parent drug itself	NR	In vivo study in cerebral ischemia rat model: Increased neuroprotective effect after prodrug administration compared to parent drug itself	NR
Ibuprofen prodrugs conjugated to ascorbic acid (147)	NR	NR	In vivo PK study in mice: 4.1 times higher AUC_0-240 min,brain_ and 7.5 times higher C_max_ of total released parent drug after i.v. administration of 48 mmol/g prodrug compared to parent drug itself K_p,brain_ of released parent drug 0.51, while for parent drug itself 0.54	NR	NR	NR
Ibuprofen prodrug conjugated to ascorbic acid (145)	In vitro study: not significant neuroprotective effect in H_2_O_2_-induced oxidative stress and injury PC12 cells treated with prodrug	NR	In vivo PK study in mice: 2.4 times higher AUC_0-480 min,brain_ and 4.2 times higher C_max_ of total released parent drug after i.v. administration of 10 mg/kg prodrug compared to parent drug itself K_p,brain_ of released parent drug 0.63, while for parent drug itself 0.34	NR	In vivo cerebral ischemia rat model: greater neuroprotective properties after i.v. administration of prodrug compared to parent drug	NR
Dual targeting to GLUTs and SVCT2						
Dual naproxen prodrug conjugated to glucose and ascorbic acid (143)	NR	NR	In vivo PK study in mice: 2.4 times higher AUC_0-480 min,brain_ and 2.9 times higher C_max_ of total released parent drug after i.v. administration of prodrug compared to parent drug itself	NR	In vivo study in cerebral ischemia rat model: Increased neuroprotective effect after prodrug administration compared to parent drug itself	NR
Dual ibuprofen prodrug conjugated to glucose and ascorbic acid (147)	NR	NR	In vivo PK study in mice: more than 4.1 times higher AUC_0-240 min,brain_ and 7.4 times higher C_max_ of total released parent drug after i.v. administration of 48 mmol/g prodrug compared to parent drug itself K_p,brain_ of released parent drug 0.25 while for parent drug itself 0.54	NR	NR	NR
Ibuprofen prodrug conjugated to glucose and ascorbic acid (145)	In vitro study: not significant neuroprotective effect in H_2_O_2_-induced oxidative stress and injury PC12 cells treated with prodrug	NR	In vivo PK study in mice: 2.6 times higher AUC_0-480 min,brain_ and 5.2 times higher C_max_ of total released parent drug after i.v. administration of 10 mg/kg prodrug compared to parent drug itself K_p,brain_ of released parent drug – 0.69, while for parent drug itself – 0.34	NR	In vivo cerebral ischemia rat model: greater neuroprotective properties after i.v. administration of prodrug compared to parent drug	NR
OCTN2						
L-carnitine prodrug of nipecotic acid (148)	NR	NR	In vivo PK study in mice: parent drug was detected in the brain 30 min after i.p. injection of 0.75 mmol/kg of prodrug, while no parent drug was detected after parent drug dosing	NR	In vivo study in mice with PTZ-induced convulsions: increase in latency of tonic convulsions after prodrug i.p. injection 75 mmol/kg, while no effect was observed after parent drug itself	NR
Glutathione transporters						
Prodrug of adamantamine conjugated to glutathione analogue (149)	NR	Inhibition of the uptake of [^3^H]glutathione transport across MDCKII cell monolayer by the prodrug	NR	NR	NR	NR
Prodrug of dopamine conjugated to glutathione analogue (149)	NR	Inhibition of the uptake of [^3^H]glutathione transport across MDCKII cell monolayer after the prodrug	NR	NR	NR	NR
Glutathione derivative of L-dopa (150)	Antioxidant activities of prodrugs in test involving Fe(II)/H_2_O_2_-induced deoxyribose degradation	NR	In vivo PK study in rats: after intragastric administration of 0.332 mmol/kg prodrugs in rats prolonged basal levels of striatal dopamine and L-dopa with lower C_max_ than after parent drug dosing	NR	In vivo study in rats: decreased locomotion and impaired grooming behaviour after 0.332 mmol/kg prodrug in rats, which was comparable to that after parent drug dosing	NR
Modified glutathione derivative of L-dopa (151)	Antioxidant activity in the DPPH-HPLC and the DMSO competition methods	NR	In vivo PK study in rats: after intragastric administration of 0.332 mmol/kg prodrug in rats increased basal levels of striatal dopamine and L-dopa	NR	NR	NR

ARPE19—Human retinal pigment epithelial cell line; AUC—Area under the drug concentration − time curve; BCH—2-amino-2-Norbornanecarboxylic Acid; DMSO—Dimethyl sulfoxide; DPPH-HPLC—2,2-diphenyl-1-picrylhydrazyl-high performance liquid chromatography; COX-Cyclooxygenase; C_max_—Maximum concentration; IC50—Concentration at which a substance exerts half of its maximal inhibitory effect; MDCKII—Madin Darby canine kidney cell line, MPTP—1,2,3,6-methyl-phenyl-tetrahydropyridin; NA – Not applicable; NR – Not reported; NMDA—*N*-methyl-D-aspartate,, 6-OHDA—6-hydroxydopamin, PK – Pharmacokinetics; Ki—Inhibitory constant; K_p,brain_—Ratio of total brain to total plasma drug concentrations; K_p,uu_,_brain_—Ratio of brain ISF to plasma unbound drug concentrations

**Table III:** Nanocarriers developed to utilize SLC transporters for improvement of brain delivery of drugs

Nanocarrier and loaded drug	Evidence of utilization of specific transporter	Information about the rate and extent of brain delivery	Information about intra-brain distribution	Pharmacodynamic evidence	Clinical evidence
LAT1					
L-dopa conjugated liposomes loaded with WP-1066 (152)	In vitro uptake study in LAT1 expressing GL261 cells: cellular uptake of liposomes was decreased when the cells were pre-treated with LAT1 antibody	L-dopa conjugated liposomes labelled with near infrared dye accumulated into mouse brain more efficiently than non-LAT1-targeted liposomes after 4 and 24 h i.v. injection in tumor bearing mice. The distribution between brain and tumor tissues was not investigated	NR	In vivo study in C57BL/6 J mice bearing orthotopic glioblastoma: L-dopa conjugated liposomes loaded with WP1066 inhibits tumor growth and enhances overall survivability in C57BL/6 J mice bearing orthotopic glioblastoma compared to non LAT1 targeted liposomes and vehicle	NR
Glutamate modified docetaxel-loaded liposomes (82)	In vitro study in C6 glioma cells: leucine and phenylalanine showed significant inhibition of the liposome accumulation into C6 glioma cells	In vivo PK study in mice: glutamate modified liposomes delivered fluorescent dye into brain in higher concentration compared to free dye and non-glutamate modified liposomes 8 h after i.v. injection in mice; docetaxel delivery was not investigated	NR	NR	NR
Phenylalanine modified solid lipid nanoparticles loaded with doxorubicin (153)	NR	In vivo PK study in rats: phenylalanine modified solid lipid nanoparticles delivered doxorubicin into brain in higher concentration compared to free doxorubicin and non-phenylalanine modified lipid nanoparticles 4 and 24 h after i.v. injection in rats	NR	NR	NR
Phenylalanine modified solid lipid nanoparticles loaded with efavirenz (154)	In vitro uptake study in BCEC and AC co-culture: competing LAT1 substrate phenylalanine decreased the permeability coefficient of the phenylalanine modified solid lipid nanoparticles through cells no statistical analysis was performed	In vivo PK study in rats: phenylalanine modified solid lipid nanoparticles increased the efavirenz percentage of the injected dose in the brain 1–24 h after i.v. injection in rats	NR	NR	NR
Glucose transporters					
Mannose, fucose and galactose coated [^3^H]-galactocerebroside labelled liposomes (155)	NR	In vivo study in mice: after i.p. injection lipids were extracted from brain, liver, kidney and spleen followed by radioactivity measurement	The distribution of the radiolabelled lipids was investigated between glia, neurons, capillaries and crude myelin	NR	NR
Mannosylated liposomes loaded with quercetin (156)	NR	NR	NR	In vivo study in rats: preservation of the activities of antioxidant enzymes and a marked inhibition of cellular oedema formation in neuronal cells in rats after i.v. injection	NR
p-aminophenyl-α-D-mannopyranoside incorporated liposomes loaded with daunorubicin (157)	In vitro study across BMVEC cells: Cellular uptake of liposomes was decreased in the presence of competitive GLUT1 and GLUT3 substrate p-aminophenyl-α-D-manno-pyranoside	NR	NR	In vivo study in C6 glioma bearing rats: daunorubicin loaded liposomes decreased the tumor size and increased the survival rats	NR
p-aminophenyl-α-D-mannopyranoside modified liposomes loaded with rhodamine (158, 159)	In vitro uptake study in in vitro BBB model: permeability of P-aminophenyl-α-D-mannopyranoside modified liposomes across in vitro BBB model was decreased by glucose transporter inhibitor cytochalasin B; cellular accumulation of the liposomes was increased in GLUT1 and GLUT3 over-expressing cells	In vivo PK study in mice: brain distribution of rhodamine loaded in P-aminophenyl-α-D-mannopyranoside modified liposomes was higher compared to free dye or non-targeted liposomes measured by whole-body imaging of mice after i.v. injection; brain distribution of rhodamine loaded in P-aminophenyl-α-D-mannopyranoside modified liposomes was decreased in mice injected with glucose transporter inhibitor phenobarbital. The brain distribution of the loaded rhodamine was investigated between different brain regions by ex vivo fluorescence imaging	The brain distribution of the loaded rhodamine was investigated between different brain regions by ex vivo fluorescence imaging	NR	NR
Ascorbic acid and glucose modified liposomes loaded with paclitaxel (160)	In vitro study in GLUT1 and SVCT2-positive C6 cells: ascorbic acid and glucose decreased the cell uptake of the dual targeted liposomes by 20%	In vivo PK study in mice: dual targeted liposomes increased the extent of brain delivery of paclitaxel in mice: K_p,brain_ of paclitaxel was 0.09, whereas the K_p,brain_ of paclitaxel loaded in the dual targeted liposomes was 0.45	NR	NR	NR
Glucose modified liposomes loaded with coumarin-6 (161)	NR	Ex vivo imaging revealed that the glucose modified liposomes increased the 1,1′-dioctadecyl-3,3,3′,3′-tetramethylindotricarbocyanine accumulation in the brain compared to conventional liposomes 1 h after i.v. injection in mice	NR	NR	NR
Dehydroascorbic acid decorated nanoparticles loaded with paclitaxel (162)	In vitro study in human-derived malignant glioma cells: cellular uptake of fluorescent dye loaded dehydroascorbic acid decorated nanoparticles was decreased in the presence of competing glucose transporter substrate, D-glucose	Fluorescent dye loaded dehydroascorbic acid decorated nanoparticles had higher brain delivery compared to nanoparticles without dehydroascorbic at 1, 2, 8 and 12 h after i.v. injection in U87 glioma bearing mice. Fluorescence microscopic imaging of glioma-bearing brain sections showed higher accumulation of the dehydroascorbic acid decorated nanoparticles into the tumor tissue compared to normal brain tissue. The brain or tumor delivery of paclitaxel was not investigated	NR	In vivo study in glioma bearing mice: dehydroascorbic acid decorated nanoparticles loaded with paclitaxel increased survival time of mice compared to that after paclitaxel treatment	NR
Glucose decorated and thiamine disulfide lock in system nanoparticles loaded with docetaxel (163)	In vitro uptake study in C6 glioma cells: cellular uptake of nanoparticles was decreased in the presence of D-glucose. Statistical significance of the decrease was not reported	In vivo PK study in mice: glucose decorated nanoparticles with or without “lock in” function increased the extent of docetaxel brain delivery compared to docetaxel in mice after i.v. injection. The K_p,brain_ were 0.89, 1.25 and 1.85 for docetaxel, docetaxel loaded in glucose decorated nanoparticles without “lock in” function and docetaxel loaded in glucose decorated nanoparticles with “lock in” function, respectively	NR	NR	NR
Dehydroascorbic acid modified micelles loaded with itraconazole (164)	In vitro uptake study in BCECs: micelle entry into cells was inhibited by competing glucose transporter substrate and inhibitor. No statistical analysis was performed	Micelles increased significantly the itraconazole plasma AUC in rats and brain concentrations in mice at 2 and 4 h after i.v. injection	NR	In vivo study in CNS infectious mice: micelles had beneficial effects over the commercial formulation in brain fungal burden recovery, survival curves and weight changes of mice	NR
SVCT2					
Ascorbic acid modified liposomes with thiamine disulfide lock in system loaded with docetaxel (165)	NR	In vivo PK study in mice: ascorbic acid modified liposomes with or without thiamine disulfide “lock in” system increased the brain delivery of docetaxel after i.v. injection in mice. The K_p,brain_ were 0.74, 1.20 and 1.52 for docetaxel, docetaxel loaded in ascorbic acid modified liposomes without “lock in” function and docetaxel loaded in ascorbic acid modified liposomes with “lock in” function, respectively	NR	NR	NR
OCTN2					
L-Carnitine-conjugated nanoparticles loaded with paclitaxel (166)	In vitro study in hCMEC/D3 cells: the cellular uptake of the nanoparticles was shown to be Na^+^-dependent L-carnitine, a competing OCTN2 substrate inhibited the uptake of nanoparticles	In vivo PK study in mice: the brain accumulation of paclitaxel was higher in mice 2, 6 and 12 h after i.v. injection of L-Carnitine-conjugated nanoparticles loaded with paclitaxel compared to injection of free paclitaxel	NR	NR	NR

BCEC—Bovine caruncular epithelial cell line; BMVEC—Bovine pulmonary microvessel endothelial cells; hCMEC/D3—Human brain endothelial capillary cell line D3; NR – Not reported; PK – Pharmacokinetics; K_p,brain_—Ratio of total brain to total plasma drug concentrations

LAT1 encoded by SLC7A5 is a functional subunit of Na^+^- and pH-independent exchanger of large branched-chain and aromatic neutral amino acids coupled with a heavy chain subunit (known as CD98 or 4F2hc, SLC3A2) (167–169). In humans, the LAT1 protein is expressed in the brain capillary endothelial cells (Table I), as well as neurons (50, 170). In rodents, the apical and basolateral localization and functional expression of the transporter at the BBB (171, 172) as well as functional expression in primary astrocytes, neurons and immortalized microglia cultures were confirmed (122, 173–175). The transporter mediates the inward flux of essential amino acids including leucine, phenylalanine, isoleucine, tryptophan, histidine and tyrosine in an antiport with tyrosine, histidine, and a non-essential amino acid glutamine (176, 177). LAT1 has been shown to be involved in the delivery of several clinically used CNS (pro)drugs including muscle relaxant baclofen, anticonvulsant gabapentin and pregabalin, antihypertensive alpha-methyldopa, anti-Parkinsonian L-dopa (178–182). However, these compounds were not specifically designed to utilize LAT1 for brain delivery; rather, their LAT-mediated transport was confirmed afterwards. LAT1 has been the most studied transporter for development of (pro)drugs and nanocarriers (Table II) to improve brain delivery of drugs (177).

Other transporters, which have been actively investigated for brain delivery of drugs are glucose transporters (GLUTs, encoded by SLC2A genes). GLUTs are responsible for the Na^+^-independent facilitated transport of glucose and other hexoses across the cellular membranes. The expression of GLUTs is cell type-specific and can be affected by disease conditions (183). High affinity GLUT1 transporter is mainly expressed at both the luminal and abluminal membranes in the brain endothelial cells of the BBB (184–186) and in the dendritic end-feet of astrocytes that enwrap brain capillaries (186, 187). The high affinity GLUT3, low-affinity GLUT2, and insulin-dependent GLUT4 have been detected in astrocytes (183). Neuronal glucose uptake is predominantly facilitated by GLUT3, although other transporters such as GLUT2 and GLUT4 may be involved in glucose uptake into neurons under specific physiological or pathophysiological conditions (183).

Several attempts have been made to utilize SVCT2, OCTN2, and glutathione transporters for transporter-mediated brain delivery of drugs (Table II,III). However, these transporters are less studied in terms of transporter-mediated drug delivery to the brain. SVCT2 is a high-affinity ascorbic acid transporter, which has been shown to be expressed at the brain endothelial cells of the BBB as well as in neurons, microglia, and astrocytes (188, 189). Moreover, functional expression of the transporter has been demonstrated in mouse brain capillary endothelial cells (188). OCTN2 is a polyspecific transporter, mediating both a Na^+^-dependent transport of L-carnitine and Na^+^-independent uptake of organic cations (60). OCTN2 is a major mediator of L-carnitine passage, which plays an important role in translocation of acetyl-moiety and contributes to the acetylcholine and acetyl-L-carnitine synthesis in the brain (190–192). The functional expression of the transporter was confirmed at the rat BBB and primary cultured brain capillary endothelial cells of rat and human (193). Moreover, the transporter protein was detected in mouse neurons, and functional expression of the transporter was shown in cultured rat cortical astrocytes (194, 195). In addition, targeting of glutathione (GSH) transporters has been used for brain delivery of drugs by prodrugs (Table II). However, in the reviewed studies, information on specific glutathione transporters which have been used for brain delivery was not clarified (149–151).

The majority of the agents for which the transporter-mediated approach has been used includes compounds with the brain intracellular targets such as: a nucleoside analogue reverse-transcriptase inhibitor zidovudine; cyclooxygenase (COX) inhibitors ketoprofen, ibuprofen, indomethacin, and naproxen; neuronal gamma-aminobutyric acid (GABA) transaminase inhibitor valproic acid, neuronal β-secretase modulator ferulic acid, antiviral efavirenz and several anti-cancer agents (Table II, III). However, the approach was also used for the improvement of brain delivery of therapeutic agents with extracellular targets including a neurotransmitter dopamine, competitive antagonist of the glycine site of the NMDA receptor 7-chlorokynurenic acid, serotonin-norepinephrine reuptake inhibitor venlafaxine and inhibitor of the GABA transporter nipecotic acid (Table II, III). However, the transporters, such as OCTN2, GLUTs, LAT1 and SVCT2 (Table II, III), used for brain delivery of mentioned therapeutic agents, are expressed at both the BBB and brain parenchymal cells, and, therefore, can be more suitable for the brain delivery of the drugs with intracellular targets. In addition, the predominant intracellular localization of metabolizing enzymes required for the release of the parent drug from prodrugs (Table II) supports the utilization of the prodrug approach for drugs with intracellular targets. Therefore, the effectiveness of the use of the transporter-mediated prodrug approach for the brain delivery of drugs with brain extracellular targets is questionable.

Importantly, the selection of the transporter for brain delivery of drugs can be affected not only by expression profiles at the BBB and brain parenchyma with regards to drug target localization, but also by the structural features of the substrates. The small size of substrates, which nutrient transporters can efficiently transport, limits the number of parent drug molecules for which the transporter-mediated prodrug delivery approach can be applied. In addition, in terms of the prodrug approach, not all drugs can be conjugated with the promoiety to be recognized as a substrate of the transporter due to the difficulties in synthesis implementation or limits of the chemical structures of the compounds. Furthermore, the availability of cost-efficient (pro)drug synthesis or nanocarrier preparation allowing high-scale production should be taken into consideration.

### Evaluation of the Mechanism of the Brain Delivery

As the main principle of the transporter-mediated approach is the use of the transporters for brain delivery of drugs, the confirmation of the utilization of the specific transporter by (pro)drugs, or nanocarriers for the BBB uptake should be confirmed. In general, the transporter-mediated passage of compounds across the cellular membrane can be investigated using several methods including *in vivo* cerebral microdialysis technique, *in situ* brain perfusion technique and PET imaging in transporter-knockout animals or using selective transporter inhibitors and substrates (112, 118, 196–198). Moreover, *in vitro* uptake experiments in brain slices or cell models with confirmed expression and function of the transporter can be used (124). Although *in vivo* and *in situ* uptake studies give insight into the transport mechanism across the intact BBB, these techniques are expensive, technically challenging, and require comprehensive design. In contrast, *in vitro* uptake studies in cellular models using transporter inhibitors and substrates give fast information about the transporter-mediated uptake of the drugs and can be used as a screening tool for a large set of compounds. In addition, the use of transporter knockout and overexpressing cell lines are a reliable tool for investigating the role of a specific transporter for the uptake of (pro)drugs and nanocarriers. Different *in vitro* BBB cell culture models including primary and immortalized brain endothelial cell models, have been developed to facilitate the investigation of drug permeation across the BBB (199). However, there is still limited information about the functional expression of the transporters in these models, which limits their use for the identification of the transport mechanism for compounds targeting transporters at the BBB.

In terms of the developed transporter-utilizing (pro)drugs, the transport mechanism has not been investigated for more than third part of (pro)drugs (Table 2). Among those studies, half of the reports investigated only the binding to the transporter, either using competitive cellular uptake assays, or *in situ* brain perfusion in rodents with the corresponding transporter substrates, i.e., amino acids, glucose, etc. However, it is important to remember that binding to the transporter does not guarantee the utilization of the transporter for cellular passage. Therefore, the uptake of the compounds in the presence and absence of the selective transporter inhibitor provides important information about the translocation of the drugs via the specific transporter across the cellular membrane. However, confirmation of the utilization of the targeted transporter for cellular uptake was performed for a limited number of (pro)drugs (Table II). In addition, several studies reported concentration-dependent cellular or BBB uptake of the developed compounds providing the data on the Michaelis–Menten transport kinetic parameters (Table II). The transport kinetic parameters, such as affinity (K_m_) and transport capacity (V_max_), are essential for understanding the capability of the (pro)drugs to utilize the transporter and planning pharmacokinetics studies. However, in some studies the kinetic parameters (K_m_ and V_max_) were calculated from linear or non-saturable cellular uptake data, which does not produce reliable results (119, 123).

The majority of the published studies on nanocarriers (Table III) lack thorough investigation of the capability of the nanocarriers to bind to the targeted transporter and evidence that the nanocarrier endocytosis or transcytosis is mediated by binding to the transporter. Commonly, transporter binding and utilization by the nanocarriers has been evaluated by the inhibitory effect of a competing substrate or inhibitor of the targeted transporter (Table 3). However, the statistical significance of the differences in drug cell accumulation or permeation across cell monolayers was not reported or the reduction in the accumulation was marginal in nine out of 16 articles listed in Table III. This may be the result of competing substrates or inhibitors lacking the sufficient affinity to prevent the nanocarrier binding to the transporter. Another explanation is that the nanocarriers do not selectively bind to the targeted transporter, and that the nanocarrier endocytosis is mediated by other mechanisms. To sufficiently determine the cell uptake mechanism, cell accumulation studies should be conducted in target transporter knockdown or knockout cells. Furthermore, localization studies of the nanocarrier-transporter complex can aid in elucidating the endocytosis mechanism and can confirm whether cell uptake of the nanocarrier is mediated by binding to the targeted transporter.

These observations highlight the importance of validation of the methods used for investigation of transporter-mediated uptake and selection of the relevant procedure fulfilling the purpose of the study. Moreover, the transporter-mediated uptake of (pro)drugs and nanocarriers *in vitro* does not provide direct evidence of the utilization of the transporter at the BBB or brain parenchyma *in vivo*. In addition, it is important to remember, that the ability to utilize the transporter does not guarantee a high extent of brain delivery, which should be evaluated in pharmacokinetic studies (79).

### Evaluation of the Brain Delivery Efficacy via Transporter-Mediated Approach

The quantitative evaluation of brain delivery efficacy via transporter-mediated approach based on the CNS pharmacokinetic principles is critical for understanding the usability of the strategy and decision-making on further development of the transporter-utilizing (pro)drug and nanocarriers. The following criteria should be investigated during the development of the transporter-utilizing (pro)drug and nanocarriers: whether (pro)drugs or nanocarriers crosses the BBB in intact form; whether (pro)drugs or nanocarriers reaches the target site in the brain (extra- or intracellular) and releases the parent drug at the target site at the adequate concentration to produce pharmacological effect. Eventually, the main determinant of brain delivery efficacy is the concentration of the active (parent) drug at site of action in the brain or an increase of the relative distribution of parent drug between the brain and other tissues after administration of prodrug or nanocarriers compared to parent drug dosing.

The brain uptake of one third of the reported (pro)drugs and nanocarriers was not investigated (Table II,III). For many developed (pro)drugs the ability to cross the BBB was shown by *in situ* perfusion techniques in rodents with a reported rate of permeation constant (K_in_). It is important to remember that the rate of BBB permeation should be differentiated from the extent of BBB permeation, which is described by K_p,uu,brain_. The rate of permeation mainly indicates whether the compound crosses the BBB and how fast this process is. Therefore, although some studies claimed improvement of drug delivery to the brain via developed prodrugs based on the higher rate of BBB permeation of prodrugs compared to parent drugs, in fact, the rate of BBB permeation cannot be used for prediction of brain delivery efficacy.

Several pharmacokinetic studies have investigated the improvement of the brain delivery extent by comparing the distribution of the released parent drug between the brain and plasma after prodrug dosing and parent drug administration in animal models (Table II). Overall, comparison of reported K_p,brain,_ AUC_u,brain_/AUC_u,plasma_ and K_p,uu,brain_ values for released parent drug after prodrug dosing and drug itself (Table II) revealed significant improvement of brain delivery extent after administration of the following prodruds/derivatives: L-lysine-methotrexate conjugate in mice (117), lysine derivative of ketoprofen in rats (112), phenylalanine derivatives of ketoprofen in mice (79), venlafaxine-thiamine disulfide-glucose prodrug in mice (144), glucose prodrug of ibuprofen in mice (145), ascorbic acid derivatives of ibuprofen in mice (145, 147). Furthermore, administration of transporter-targeted nanocarriers of paclitaxel (160) and docetaxel (163, 165) in mice increased the K_p,brain_ of released parent drugs compared to that of after dosing of the drugs themselves. However, in the case of delivery of anti-cancer agents via L-lysine-methotrexate conjugate as well as paclitaxel and docetaxel nanocarrier formulations, the extent of drug delivery into tumors would be a more relevant parameter to investigate than K_p,brain_.

Importantly, one should remember, that K_p,brain_ and AUC_u,brain_/AUC_u,plasma_ provide information about the distribution of the parent drug in the whole brain including both intra- and extracellular space compared to plasma, while K_p,uu,brain_ gives knowledge about the distribution of the parent drug between ECF and plasma. As the target can be located in intracellular compartment of the brain parenchyma, the main determinant of successful application of the approach for delivery of drugs with the brain parenchymal intracellular targets is an improvement of unbound steady-state concentrations of released parent drug in intracellular compartment in comparison to parent drug dosing. Therefore, the investigation of the intra-brain distribution and prediction of the intracellular brain parenchymal concentration of the drug or released parent drug from prodrug or nanocarriers is crucial. In this respect, the contribution of parent drug released in plasma and entered the brain should be investigated to confirm the fact that the bioconversion occurs inside the brain. Among the reviewed studies, only two reports investigated intra-brain distribution of the released parent drug after prodrug dosing. In cerebral microdialysis study in rats, significantly higher brain intracellular distribution of ketoprofen was reported after LAT1-utilizing derivative of ketoprofen compared to parent drug dosing (112). Later, another derivative of ketoprofen was reported to improve intracellular brain parenchymal delivery of the parent drug in mice, as investigated by a combinatory mapping approach (124). Our review highlights the importance of thorough evaluation of drug delivery efficacy via a transporter-mediate approach not only to the brain, but to the target site inside the brain.

#### Drug Delivery to the Brain in Disease Conditions

Recent studies have revealed the breakdown and dysfunction of the BBB and NVU in several CNS diseases (200, 201). Transporter expression and function can be changed in neuropathological conditions, which can result in altered brain delivery efficacy of drugs. Protein expression of several SLC transporters was reported to be altered at the BBB of AD patients (52, 202), which can affect the BBB permeation of drugs, substrates of these transporters. In addition, brain tumors are known to have disrupted BBB due to the tumor vasculature becoming increasingly heterogeneous as the tumor grows (203). The rapid growth of cancer cells leads to increased requirements for blood flow at the tumor site and the subsequent abnormal angiogenesis has been shown to compromise BBB integrity (204). As a result, larger drug concentrations at the tumor site compared to healthy brain, have been observed in both preclinical brain cancer tumor models and in cancer patients (205). However, BBB integrity can differ between different tumor sites as described by Lockman *et al.* (2010) in the study in mice with brain metastases of breast cancer (206). Therefore, in order to gain sufficient drug concentrations throughout brain tumors, efficient drug delivery strategies are needed. The expression of nutrient transporters, such as GLUT1 and LAT1, has been shown to be high in both, the BBB and in glioma (207, 208), which led to the development of different transporter-utilizing anti-cancer drug delivery strategies in order to reach the cancer cells residing beyond the intact BBB (Table II). However, the targeting strategy has to be selected carefully as it has been shown that the expression of nutrient transporters, such as GLUT1, in the intra-tumor microvasculature can differ between cancer subtypes, as it was shown by Yonemori *et al*. (2010) in brain metastases of triple negative and HER2/neu-positive breast cancers (209).

None of the reviewed studies quantitatively investigated the transporter-mediated brain or tumor delivery efficacy of the drugs at the target site in *in vivo* disease models. This aspect needs to be investigated in the future, as drug delivery to the target site in healthy and diseased brains can differ significantly.

#### Evaluation of Pharmacodynamic Response Improvement due to Increased Drug Delivery

The final aim of the transporter-mediated drug delivery approach is to increase the concentration of the delivered drug at the target site in the brain, resulting in higher pharmacological response. Therefore, investigation of the improvement of pharmacological effect of the drug after transporter-mediated (pro)drug or nanocarrier administration *in vivo* in disease models provides important information about the success of the drug delivery. Although prodrugs are considered to be inactive, their pharmacological activity should be investigated at the early stages of prodrug development. Importantly, the pharmacological activity of the majority of prodrugs has not been reported (Table II). Among the developed prodrugs, the lack of activity was confirmed only for D-glucose-conjugated prodrug of 7-chlorokynurenic acid (133, 134), glycosyl prodrugs of dopamine (135) and ibuprofen prodrugs (145). In contrast, derivatives of zidovudine (127, 128), glutathione conjugate of L-dopa (126), modified glutathione derivative of L-dopa (151), dopamine-gluconamine and gluconamide (140) as well as ketoprofen derivatives (113) demonstrated pharmacological activity and should be considered as analogues. Moreover, the improvement of pharmacological effect in disease models *in vivo* by prodrug approach was shown only for the prodrug of 7-chlorokynurenic acid (133, 134) and ibuprofen prodrugs (145) when compared to parent drug administration. Importantly, the increase in pharmacological efficacy after ibuprofen prodrug dosing could be explained by higher drug delivery of ibuprofen to the brain in prodrug form compared to the parent drug itself (145). However, transporter-mediated uptake of both the prodrugs of 7-chlorokynurenic acid and ibuprofen were not reported. Overall, limited studies investigated the improvement of pharmacological efficacy in animal models after dosing of the derivative (Table II). Although pharmacological efficacy of some drugs was claimed to be improved after administration of developed derivatives compared to parent drug dosing, in these studies, the derivatives as such were either shown to be active or their activity has not been investigated.

The *in vivo* efficacy of the nanocarrier-delivered drug was compared to the drug itself in disease models in five out of 16 studies listed in Table III. From these five studies, in two, *in vivo* delivery into the target tissue, brain or brain tumor, was evaluated in any capacity. Shao *et al.* (2014) showed in U87 xenograft glioma mouse model that the loading of paclitaxel into dehydroascorbic acid-decorated micelles increased the drug efficacy (162). However, in studies investigating the tumor delivery of the micelles, instead of the anti-cancer drug paclitaxel, the micelles were loaded with a fluorescent dye. The enhanced tumor delivery of the fluorescent dye via the micelles combined with the *in vivo* efficacy results make it compelling to conclude that the micelles enhanced the tumor delivery of paclitaxel, leading to an improved *in vivo* efficacy. However, due to the lack of pharmacokinetic data for paclitaxel, such conclusions cannot be made. In another study, Shao *et al.* (2015) reported dehydroascorbic acid modified micelles loaded with itraconazole, which enhanced the antifungal efficacy of itraconazole in the CNS infectious mouse model (164). The brain itraconazole brain delivery was investigated at only two time points, 1 and 4 h, after the i.v. injection in mice. Therefore, although the drug concentration was significantly higher in micelle-injected mice compared to commercial itraconazole injection, the greater antifungal efficacy of the micelle formulation due to higher extent of brain drug delivery cannot be concluded. In conclusion, the transporter-targeting nanocarriers show promise in drug delivery into the brain and brain tumors. However, more thorough pharmacokinetic studies should be conducted in order to prove that enhanced *in vivo* efficacy is resulted by higher extent of the drug delivery into the target tissue.

#### Effect on Transporter Protein Expression and Function

For delivery via a transporter-mediated brain targeting approach, (pro)drugs and nanocarriers should possess high affinity to the transporter, which can affect the passage of physiologically important transporter substrates (210). The possibility that the developed transporter-utilizing (pro)drugs and nanocarriers can interfere with the homeostasis of endogenous substrates of the transporters should be investigated to address safety issues. Limited studies have addressed this issue. In Puris et al. (2019), we observed no impact of ketoprofen prodrug on amino acid exchange and LAT1 protein expression using *in vitro* brain slice technique (124), although the findings of this study need to be confirmed *in vivo*. Nanocarriers have been shown to have a transient effect on targeted transporter plasma membrane localization as, after binding, the nanocarrier-transporter complex is internalized in the cell followed by recycling of the transporter back on the plasma membrane (82). This is expected as the endocytosis or transcytosis of the nanocarrier is based on the internalization of the protein-nanocarrier complex. Future research should focus on the investigation of the effect of transporter-utilizing (pro)drugs and nanocarriers on transporter function and expression.

#### Translation from Preclinical Models to Human

Finally, none of the reported transporter-utilizing (pro)drugs or nanopartilce/liposomes has proceeded to clinical trials. However, differences in transporter and enzyme expression and function in preclinical models and humans may subsequently lead to interspecies variations in brain delivery of (pro)drugs and nanocarriers and the release of parent drug. Uchida *et al.* (2011) revealed significant interspecies differences in transporter protein expression of the BBB transporters (50). In order to predict the effectiveness of the transporter-utilizing (pro)drugs and nanocarriers in human, the future studies should shed light on the understanding of interspecies differences in the tissue-specific protein expression and function of the transporter as well as enzyme(s) responsible for the bioconversion of the prodrugs.

## Conclusions

The present review provides a systematic up-to-date summary of progress in development of transporter-utilizing (pro)drugs and nanocarriers for brain delivery of drugs. We revealed, that despite many attempts, which have been made to develop transporter-utilizing (pro)drugs and nanocarriers for brain delivery of different drugs, there are several factors limiting the rational development and successful application of the approach to humans. First, the mechanism of transporter-mediated prodrug transport across the BBB is not investigated thoroughly in the majority of studies. Especially, prodrug binding to intracellular binding site of the abluminal BBB transporter and prodrugs’ efflux mechanism to brain parenchyma remains unstudied. In the case of transporter-mediated nanocarrier delivery, binding to the targeted transporter should be more thoroughly investigated. In addition, the transport processes across the BBB into target cells within the brain parenchyma are complex, and the optimal utilization of the drug delivery strategy will require more research. Especially, the detailed knowledge of the nanocarrier-transporter complex internalization and following sorting processes and exocytosis would enable rational design of nanocarriers capable of efficient transcytosis across the BBB by minimizing the lysosomal degradation of the nanocarrier in the endothelial cells. This, in turn, can enable delivery of drug cargo in the target cell within the brain parenchyma. Second, information about enzymatic conversion of prodrugs at the BBB and within the brain is not known. Third, the main limiting factor is that the main CNS pharmacokinetic principles are not followed during the evaluation of drug delivery efficacy of nanocarriers and (pro)drugs, which leads to inaccurate interpretation of the results or inadequate investigation of the brain delivery of drugs. Thus, in order to achieve the desirable therapeutic effect and adequate safety of the drugs delivered to the brain via transporter-mediated approach, the focus should be on the thorough investigation of utilization of transporter for cellular uptake and detailed evaluation of the efficacy of drug delivery into the brain and within the brain. All in all, although the transporter-mediated drug delivery approach can provide tempting opportunities for drug delivery to the brain, it is currently difficult to assess the potential of the approach due to the lack of pharmacokinetic evaluation of the prodrugs and nanocarriers. Therefore, the strategy requires more thorough mechanistic and pharmacokinetic investigation in order to be used for brain delivery of therapeutics in humans.

### Conflict of Interest

The authors declare no competing financial interest.

## Abbreviations

*ABC*: ATP binding cassette
*AMT*: Absorption-mediated transport
*ARPE19*: Human retinal pigment epithelial cell line
*ASCT*: Alanine/Serine/Cysteine-preferring transporter
*AUC*: Area under the total concentration − time curve
*BBB*: Blood–brain barrier
*BCEC*: Bovine caruncular epithelial cell line
*BCH*: 2-Amino-2-Norbornanecarboxylic Acid
*BCRP*: Breast cancer resistance protein
*BCSFB*: Blood–cerebrospinal fluid barrier
*BGT*: Sodium- and chloride-dependent betaine transporter
*BMVEC*: Bovine pulmonary microvessel endothelial cells
*BUI*: Brain uptake index
*CB*: Cellular barrier
*Cl*: Clearance
*CMT*: Carrier-mediated transport
*CNS*: Central nervous system
*COX*: Cyclooxygenase
*C*_*max*_: Maximum concentration
*C*_*u, cell*_: Unbound concentrations of drugs inside the brain parenchymal cells
*CSF*: Cerebrospinal fluid
*DMSO*: Dimethyl sulfoxide
*DPPH-HPLC*: 2,2-Diphenyl-1-picrylhydrazyl-high performance liquid chromatography
*EAAT*: Excitatory amino acid transporter
*ECF*: Extracellular fluid
*ENT*: Equilibrative nucleoside transporter
*FATP*: Fatty acid transporter protein
*LCFA*: Long-chain fatty acids
*hCMEC/D3*: Human brain endothelial capillary cell line D3
*GABA*: Gamma-aminobutyric acid
*GLUT*: Glucose transporter
*IC50*: Concentration at which a substance exerts half of its maximal inhibitory effect
*ISF*: Brain interstitial fluid
*K*_*i*_: Inhibitory constant
*K*_*in*_: Unidirectional transfer constant
*K*_*m*_: Substrate concentration at which the reaction rate is half of its maximal value
*K*_*p*_: Ratio of total concentrations in the brain and plasma
*K*_*p*__*,uu,brain*_: Ratio of unbound concentrations in the brain ISF and plasma
*K*_*p*__*,uu,cell*_: Unbound drug concentration ratio in intra- and extracellular compartments of the brain
*LAT1*: Large neutral amino acid transporter
*MCT*: Monocarboxylic acid transporter
*MDCKII*: Madin Darby canine kidney cell line
*MDR1*: Multidrug resistance protein 1
*MPTP*: 1,2,3,6-Methyl-phenyl-tetrahydropyridin
*MRP*: Multidrug resistance-associated protein
*NMDA*: *N*-Methyl-D-aspartate
*NVU*: Neurovascular unit
*OAT*: Organic anion transporter
*OATP*: Organic-anion-transporting polypeptide
*OCT*: Organic cation transporter
*OCTN2*: Organic cation / carnitine transporter 2
*6-OHDA*: 6-Hydroxydopamin
*PET*: Positron emission tomography
*P-gp*: P-glycoprotein
*PK*: Pharmacokinetics
*PS*: Permeability surface area product
*RFC*: Reduced folate carrier
*rT3*: Reverse triiodothyronine
*SLC*: Solute carrier
*SPECT*: Single-photon emission computed tomography
*SUR1*: Sulfonylurea receptor
*SVCT2*: Sodium-dependent vitamin C transporter 2
*T3*: Triiodothyronine
*T4*: Thyroxine
*TJ*: Tight junction
*V*_*brain ISF*_: Volume of distribution in brain interstitial fluid
*VLCFA*: Very long-chain fatty acids
*V*_*max*_: Maximal velocity
*V*_*u*__*,brain*_: Unbound volume of distribution in the brain
*V*_*u, cell*_: Unbound volume of distribution in the brain parenchymal cells
